# Pigment Epithelium‐Derived Factor Deficiency Impairs Hippocampal Glutamate Homeostasis and Cognitive Function by Downregulating Astrocytic GLT‐1

**DOI:** 10.1002/advs.202500402

**Published:** 2025-09-14

**Authors:** Jin‐Hui Shi, Qi‐Long Tang, Jin‐Hong Wang, Yan‐Lan Long, Sai‐Feng Zhao, Zhen Zhao, Wan‐Ting Xie, Zi‐Ming Li, Hao‐Ming Lu, Tian‐Xiao Gao, Zhen‐Zhen Fang, Ti Zhou, Bo‐Xing Li, Xia Yang, Guo‐Quan Gao, Wei‐Wei Qi

**Affiliations:** ^1^ Department of Biochemistry and Molecular Biology, Zhongshan School of Medicine Sun Yat‐sen University Guangzhou 510080 China; ^2^ Guangdong Province Key Laboratory of Brain Function and Disease, Zhongshan School of Medicine Sun Yat‐sen University Guangzhou 510080 China; ^3^ Guangdong Provincial Key Laboratory of Diabetology Guangzhou Guangdong 510630 China; ^4^ Key Laboratory of Human Microbiome and Chronic Diseases (Sun Yat‐sen University), Ministry of Education Guangzhou 510655 China; ^5^ Guangdong Engineering & Technology Research Center for Gene Manipulation and Biomacromolecular Products (Sun Yat‐sen University) Guangzhou 510080 China; ^6^ China Key Laboratory of Tropical Disease Control (Sun Yat‐sen University), Ministry of Education Guangzhou 510080 China; ^7^ Department of Neurology, Sun Yat‐Sen Memorial Hospital Sun Yat‐sen University Guangzhou 510120 China; ^8^ Department of Neurology, Chaozhou People’s Hospital Chaozhou 521011 China; ^9^ Department of Nuclear Medicine, Sun Yat‐sen University Cancer Center, State Key Laboratory of Oncology in South China, Collaborative Innovation Center for Cancer Medicine Guangzhou 510060 China

**Keywords:** alzheimer's disease (AD), glutamate homeostasis, glutamate transporter‐1 (GLT‐1), pigment epithelium‐derived factor (PEDF)

## Abstract

Maintenance of glutamate homeostasis is essential for synaptic plasticity and cognition. Disrupted glutamate‐glutamine cycling causes chronic excitotoxicity, a key driver of cognitive deficits in Alzheimer's disease (AD), though regulatory mechanisms remain unclear. Pigment epithelium‐derived factor (PEDF), a neuroprotective protein declining with age, is demonstrated here to play a novel role in synaptic glutamate clearance. Analysis of peripheral blood samples from 19 patients with AD and 75 non‐dementia control subjects revealed lower levels of PEDF in patients, and loss of PEDF correlates with cognitive decline. PEDF‐deficient mice exhibit defective learning and memory, and higher susceptibility to AD. Furthermore, PEDF deficiency impaired synaptic plasticity and dendritic spine morphology. Mechanistically, PEDF inhibits ubiquitin‐proteasome‐dependent degradation of astrocytic glutamate transporter‐1 (GLT‐1) and normally guarantees elimination of synaptic glutamate by modulating the protein kinase C signaling pathway. Strikingly, restoring PEDF rescued cognitive deficits in a mouse model of AD, and upregulation of GLT‐1 rescued cognitive impairment in PEDF‐deficient mice. Collectively, these findings reveal PEDF is a physiologic regulator of synaptic glutamate homeostasis. Targeting PEDF deficiency‐induced neural impairment may provide a novel avenue for the development of new therapeutic applications for neurodegenerative diseases associated with glutamate‐induced excitotoxicity.

## Graphical abstract

The chronic glutamate‐induced excitotoxicity hypothesis has profoundly informed the therapeutic strategies employed in Alzheimer’s disease (AD). This study shows pigment epithelium‐derived factor (PEDF) regulates astrocytic glutamate transporter‐1 (GLT‐1)‐mediated glutamate homeostasis and cognition. Reduced PEDF correlates with lower Mini‐Mental State Examination (MMSE) and Montreal Cognitive Assessment (MoCA) scores. PEDF preserves astrocytic glutamate uptake, offering mechanistic insights into AD progression.

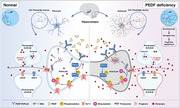

## 1 Introduction

Cognitive function involves a series of psychologically and socially based behaviors, including learning memory and spatial orientation, that are crucial for survival.^[^ ^1^ ^]^ Alzheimer's disease (AD) is a highly heterogeneous aging‐related disease, and the most frequent cause of cognitive decline.^[^ ^2^ ^]^ Aberrant glutamate‐induced excitability is a significant risk factor for pathological changes in the early stage of AD.^[^ ^3^ ^]^ Disruption of glutamate homeostasis causes a variety of progressive neurologic disorders.^[^ ^4^ ^]^ Glutamate homeostasis refers to the tightly regulated balance of glutamate levels in the extracellular space to ensure optimal synaptic function while preventing excitotoxicity. This balance is maintained by coordinated processes such as glutamate release from presynaptic neurons, uptake by glial cells and neurons (primarily through excitatory amino acid transporters), and the synthesis and recycling of glutamate. Disruption in glutamate homeostasis can lead to either excessive accumulation, contributing to neurotoxicity, or insufficient glutamate levels, impairing synaptic transmission.^[^ ^5^ ^]^ Glutamate homeostasis represents the balance between glutamate release and elimination and depends on neuron‐glia interactions.^[^ ^6^ ^]^ Astrocytes, the majority of glial cells, ensheath most excitatory synapses and help to regulate neuronal transmission.^[^ ^7^ ^]^ Astrocytes express high levels of glutamate transporters in their end‐feet, and thus help to balance the synaptic glutamate concentration in the brain and generally maintain glutamate homeostasis between synapses.^[^ ^8^ ^]^ Astrocytic uptake of glutamate (as a consequence of astrocytic activity) has been shown to actively modulate neuronal excitability and synaptic transmission, and plasticity.^[^ ^9^ ^]^ Glutamate transporter 1 (GLT‐1; or excitatory amino acid transporter 2, EAAT2), the major glutamate transporter protein in the brain, is almost exclusively expressed by astrocytes and accounts for ≈90% of functional uptake of extracellular glutamate.^[^ ^10^, ^11^ ^]^ A smaller fraction of GLT‐1 is expressed in neurons, specifically in axon terminals.^[^ ^12^, ^13^, ^14^ ^]^ The majority of GLT‐1 protein is located in astrocytes, where it plays a crucial role in glutamate uptake and maintaining proper brain function.^[^ ^15^, ^16^ ^]^ Expression of GLT‐1 has been reported to be significantly reduced in the brains of elderly individuals and patients with AD.^[^ ^17^ ^]^ However, the molecular mechanisms that underlie the regulation of astrocytic GLT‐1 are not yet fully understood.

Accumulating evidence indicates the serine protease inhibitor (SERPIN) superfamily plays essential roles in the onset and progression of multiple neurodegenerative disease.^[^ ^18^, ^19^, ^20^ ^]^ A study of 708 prospectively collected autopsied brains revealed lower serine protease inhibitor F1 (*SERPINF1*) levels in subjects with AD and individuals who were pre‐symptomatic,^[^ ^21^ ^]^ suggesting that *SERPINF1* deficiency may be an essential feature associated with the onset and progression of AD. Given this notable association between decreased *SERPINF1* expression and the burden of AD pathology, it is plausible to suggest that *SERPINF1* deficiency may be a risk factor for the onset of AD. The gene *SERPINF1*, which encodes the secreted glycoprotein pigment epithelium‐derived factor (PEDF), was initially described as a neurotrophic factor and is a non‐inhibitory member of the SERPINs superfamily.^[^ ^22^ ^]^ PEDF was originally identified in retinal pigment epithelial cells. An analysis of the adult human brain and spinal cord showed significant levels of the PEDF transcript in most regions of the nervous system.^[^ ^22^ ^]^ Ventricular membrane cells may be an essential source of PEDF in human cerebrospinal fluid (CSF), suggesting that a substantial portion of the brain may be impregnated with it.^[^ ^23^ ^]^ PEDF has been demonstrated to play critical roles in neuroprotection,^[^ ^24^ ^]^ development,^[^ ^25^ ^]^ stem cell differentiation,^[^ ^26^ ^]^ and neurogenesis.^[^ ^27^ ^]^ PEDF has been reported to possibly play an essential role in activating microglial metabolism while blocking proliferation, and soluble factors released by PEDF‐stimulated microglia similarly block the proliferation of astrocytes.^[^ ^28^ ^]^ Moreover, PEDF exerts anti‐inflammatory properties, inhibiting the activation of astrocytes and reducing the secretion of pro‐inflammatory cytokines such as TNF‐α and IL‐1β, thereby reducing the inflammatory response.^[^ ^29^ ^]^ It is worth noting that overexpression of PEDF significantly reduced the glutamate concentration in the hippocampus of chronic unpredictable mild stress models of depression.^[^ ^30^ ^]^ Although PEDF has been found to exhibit neuroprotective effects against excitotoxicity, the majority of studies on the mechanism of PEDF are restricted to cell culture or animal model systems under pathological conditions.^[^ ^23^, ^31^ ^]^ Therefore, the physiological functions of PEDF in the nervous system, especially in astrocytes, are still poorly understood.

Previous studies have demonstrated that PEDF expression declines with age.^[^ ^32^, ^33^ ^]^ Similarly, lower serum and hippocampal PEDF levels were observed in aged C57BL/6 mice.^[^ ^32^ ^]^ However, the mechanisms by which PEDF exerts neuroprotective effects have not been thoroughly elucidated. Here, we reveal previously unknown roles for PEDF in cognitive function through the regulation of glutamate homeostasis. Our results suggest, for what we believe is the first time, that PEDF can protect hippocampal pyramidal neurons from excitotoxicity by regulating the levels of astrocytic GLT‐1 and indicate that downregulation of PEDF plays an essential role in the development and progression of AD. We demonstrate that restoring PEDF expression to prevent astrocytic GLT‐1 degradation may represent a promising therapeutic strategy for AD and related neurodegenerative disorders.

## 2 Results

### 2.1 PEDF is Downregulated in the Hippocampus of Patients with AD and Mouse Model of AD

**Figure 1:**
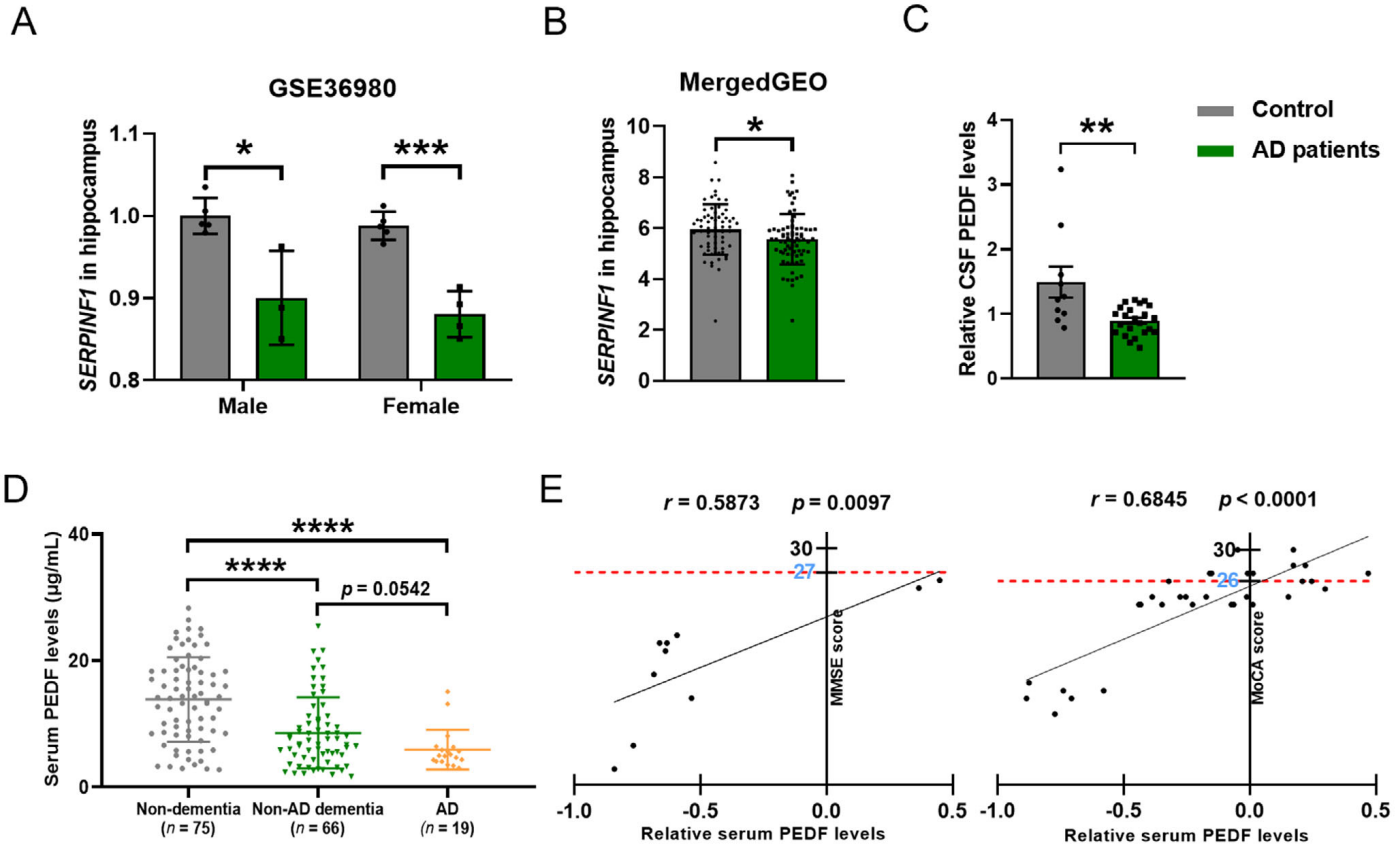
PEDF is decreased in the hippocampus of AD patients. A), The relative *SERPINF1* mRNA level in the hippocampus of AD patients (3 males and 4 females) and healthy controls (5 males and 5 females), the dataset was obtained from GSE36980. B), The normalized *SERPINF1* transcripts in the hippocampus of AD patients (*n* = 66) and healthy controls (*n *= 74), the dataset was merged from GSE28146, GSE29378, GSE36980, GSE48350, and GSE5281. C), PEDF levels in CSF from a cohort of the large‐scale CSF proteomic analyses (https://doi.org/10.7303/syn20933797). AD patients (*n* = 23), control (*n* = 10). D), PEDF concentration in peripheral blood from AD patients (*n* = 19), non‐AD dementia patients (*n* = 66), and non‐dementia control (*n* = 75) subjects. E), Correlation of serum PEDF level with MMSE score, and MoCA score; Pearson's correlation analysis (Two‐sided). All data are presented as mean ± S.E.M. One‐way ANOVA combined with Tukey's multiple comparison (D); Comparison by two‐tailed unpaired Student's *t*‐test unless otherwise indicated. ^*^ *p* < 0.05, ^**^ *p* < 0.01, ^***^ *p* < 0.001, ^****^ *p* < 0.0001; n.s., not significant. See the source data for the statistical details.

First, we evaluated the relationship between PEDF and AD. Cross‐platform normalized analysis of *SERPINF1* mRNA levels using the AlzData database revealed that *SERPINF1* mRNA was expressed at significantly lower levels in various regions of the brain in patients with AD compared to age‐matched healthy control subjects (Figure S1A, Supporting Information). Moreover, analysis of RNA sequencing (RNA‐seq) datasets (GSE36980) published in the NCBI's Gene Expression Omnibus (GEO) database also showed that *SERPINF1* was reduced in the hippocampus of AD patients compared to healthy controls (**Figure** **1**A). Furthermore, merged analysis of multiple GEO databases (GSE28146, GSE29378, GSE36980, GSE48350, and GSE5281) also revealed reduced expression of *SERPINF1* in the hippocampus of patients with AD (Figure 1B; Figure S1B, Supporting Information). Notably, analysis of a cohort from a reported CSF proteomics study showed a significant decrease in PEDF level in the CSF of AD patients compared to the control (Figure 1C).

Next, we examined the levels of PEDF in peripheral blood from 66 patients with non‐AD dementia (30 males and 36 females), 19 patients with AD (7 males and 12 females), and 75 non‐dementia control subjects (34 males and 41 females). The demographic and clinical characteristics of the subjects are listed in Table S1 (Supporting Information). PEDF levels were significantly lower in patients with non‐AD dementia and AD than in the non‐dementia subjects (Figure 1D). Moreover, lower serum PEDF negatively correlated with the Mini‐Mental State Examination (MMSE) or Montreal Cognitive Assessment (MoCA) cognitive scale scores of the patients with dementia and AD (Figure 1E).

PEDF is ubiquitously expressed in the adult mouse brain, including the cerebellum (CB), medulla (MY), thalamus (TH), olfactory cortical areas (OLF), and hippocampus (HPF) (Figure S1C,D, Supporting Information). Western blotting (WB) showed that PEDF was significantly downregulated in the hippocampus of 6‐month‐old APP/PS1 mice and 8‐week‐old 5 × FAD model mice compared to wild‐type controls (Figure S1E,F, Supporting Information). Taken together, these data indicate that decreased PEDF levels may be a risk factor for AD.

### 2.2 PEDF Deficiency Results in Learning and Memory Defects

**Figure 2:**
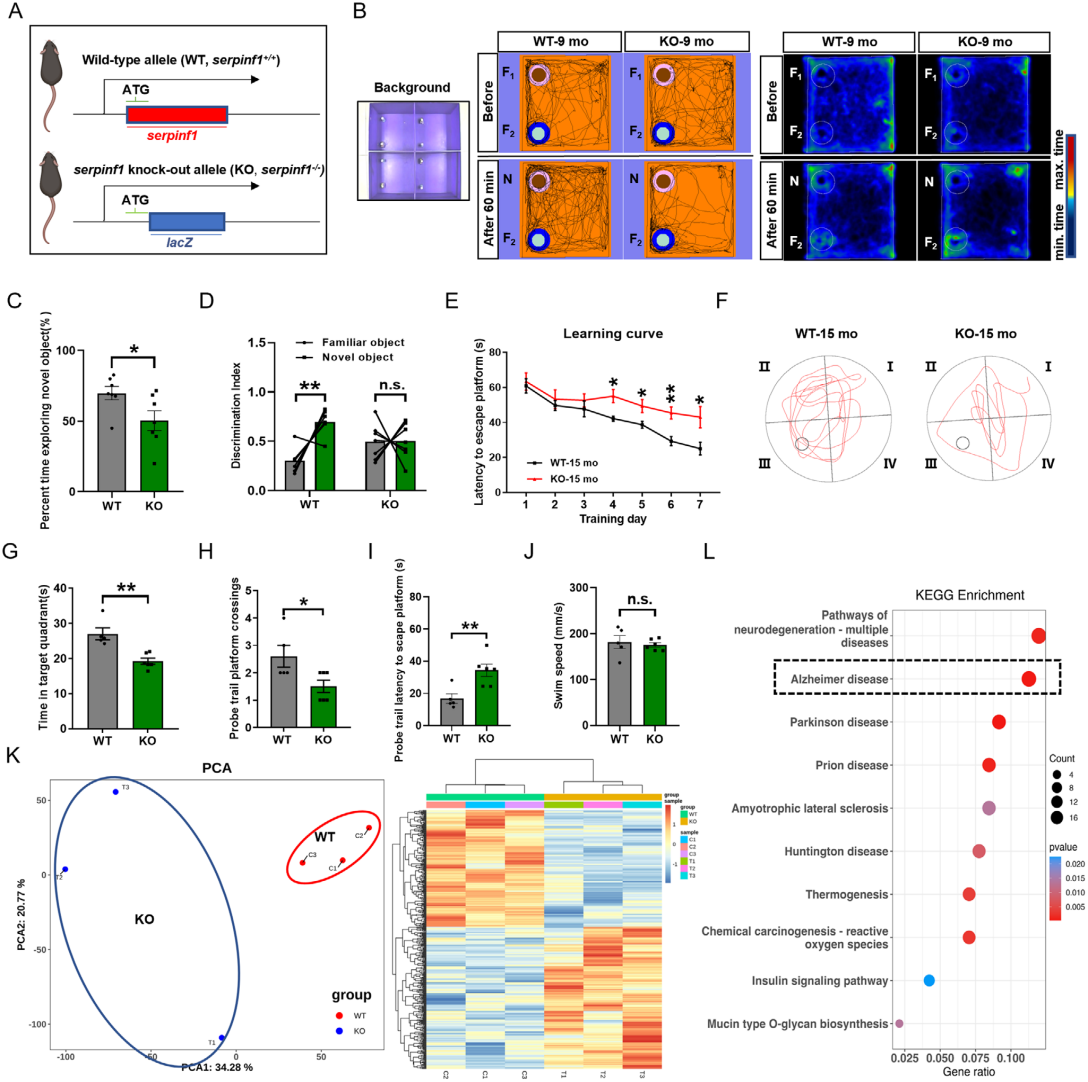
Deletion of PEDF impairs learning and memory in mice. A), Overview of the strategy to generate *serpinf1* ^−/−^(KO) mice. B), Representative track and heat map images by 9‐mo‐old WT and KO mice in the NOR test. C), Quantification of percentage time spent in the novel and familiar object during the test phase (*n* = 7 mice per group). D), The discrimination index is calculated as the ratio of interaction time with the novel or familiar object to total interaction time with the novel object and familiar object. Two‐tailed paired Student's *t*‐test. E), Latency to reach the hidden platform during spatial training in the Morris water maze. Two‐way ANOVA with Bonferroni's multiple comparison. F), Representative track images by 15‐mo‐old WT and KO mice in the probe trial of the MWM test. G–J), Time spent in target quadrant G), platform crossing count H), latency of first time to enter the initial platform I) and mean swimming speed J) in the probe trial of the MWM test (*n* = 5 mice in WT group and *n* = 6 mice in KO group). K), PCA analysis and heat map of DEGs in the hippocampus of KO and WT groups (*n* = 3 per group). L), KEGG enrichment analysis of DEGs. All data are presented as mean ± S.E.M. Comparison by two‐tailed unpaired Student's *t*‐test unless otherwise indicated. ^*^ *p* < 0.05, ^**^ *p* < 0.01; n.s., not significant. See the source data for the statistical details.

To investigate the potential effects and AD‐related phenotypes related to the deletion of *SERPINF1*, we generated a global *serpinf1*‐deletion mouse line (**Figure** **2**A). *Serpinf1* knockout (KO) mice exhibited normal body sizes and brain weights (Figure S2A,B, Supporting Information). After verifying that hippocampal PEDF levels were almost wholly reduced (Figure S2C–F, Supporting Information), we investigated the behavioral performance of the KO mice and WT controls. No differences were observed in general locomotion in the open field test (OFT) (Figure S2G–M, Supporting Information). The KO mice displayed an object recognition deficit at 9 months‐of‐age in the novel object recognition (NOR) test (Figure 2B–D), but did not exhibit spatial memory deficits at 12 months‐of‐age in the Morris water maze (MWM) or fear conditioning (FC) (Figure S2N–U, Supporting Information). However, the KO mice showed a spatial reference learning deficit in the MWM and Y‐maze task at 15 months‐of‐age (Figure 2E‐J; Figure S3A–D, Supporting Information) and impaired performance in the MWM and Y‐maze task at 18 months of age (Figure S3E–N, Supporting Information).

Next, principal component analysis (PCA) of RNA‐seq data indicated significant differences in the gene expression patterns of hippocampal tissues from 9‐month‐old KO and WT mice. These differences were further supported by the clustering heatmap of differentially expressed genes (DEGs) (Figure 2K). Notably, Kyoto Encyclopedia of Genes and Genomes (KEGG) and Gene‐set enrichment analysis (GSEA) pathway enrichment analysis of the DEGs revealed high enrichment of genes and pathways involved in neurodegenerative diseases, with Alzheimer's disease as the top‐ranked enriched disease type (Figure 2L; Figure S3O, Supporting Information). GSEA analysis within the biological process (BP) category of Gene Ontology (GO) pathway analysis indicated significant suppression of terms related to cognitive function, as well as learning and memory (Figure S3P, Supporting Information). Subsequently, the top 50 proteins encoded by DEGs were subjected to protein‐protein interaction (PPI) network functional enrichment analysis using the STRING database, and further KEGG pathways enrichment analysis showed that these proteins were significantly enriched for the development of AD (Figure S3Q and Table S2, Supporting Information). Moreover, the gene expression dataset of hippocampus from AD patients (GSE48350) was used for comparison with the DEGs identified in the current study (Figure S3R, Supporting Information). Together, these data indicate PEDF deficiency‐induced age‐related cognitive decline and an AD‐like phenotype.

### 2.3 PEDF Bidirectionally Regulates the Progression of Cognitive Deficits in a Mouse Model of AD

**Figure 3:**
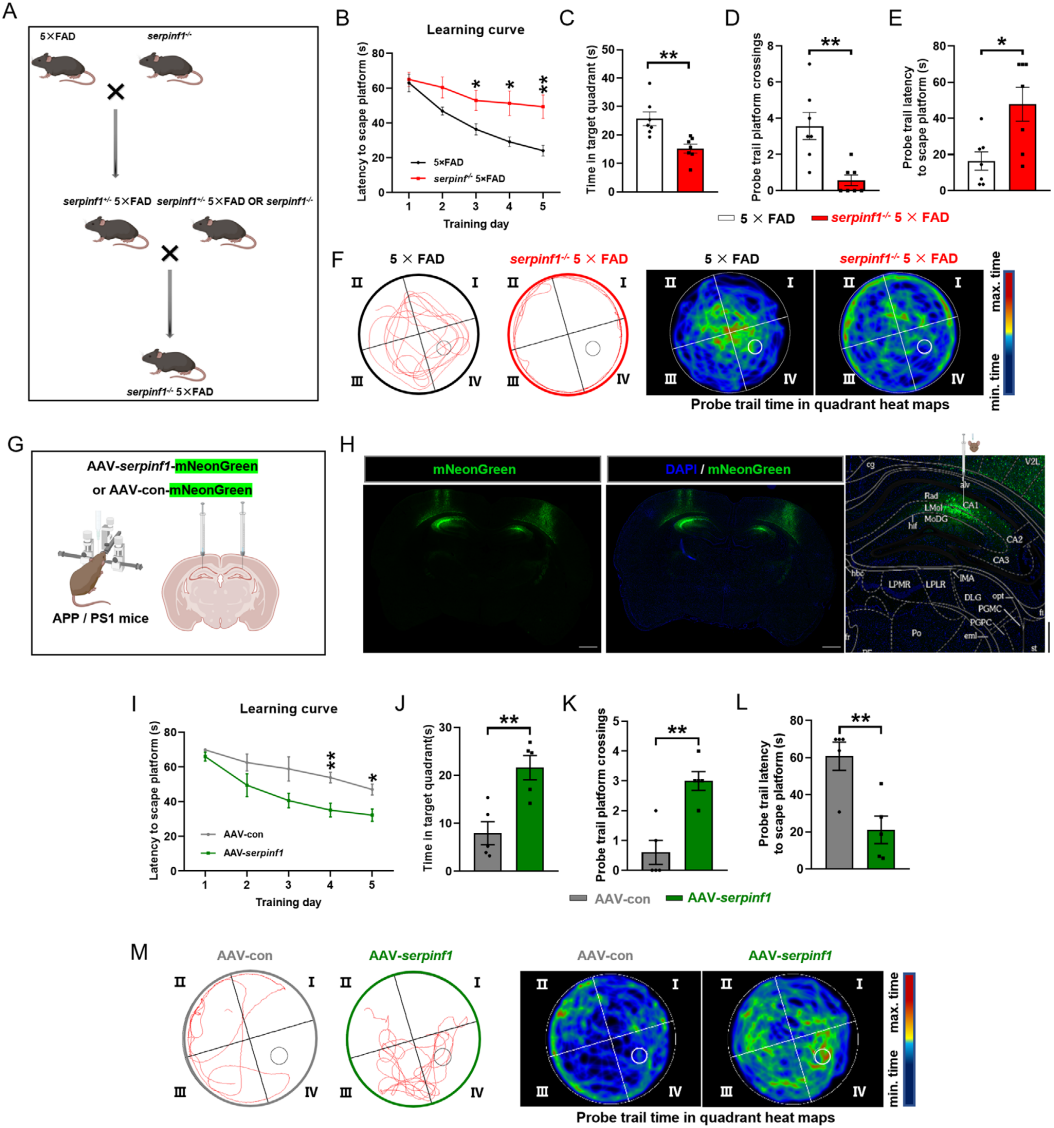
PEDF bidirectionally regulates cognitive deficits in mouse model of AD. A), Schematic representation of breeding strategies in the *serpinf1* ^−/−^ 5 × FAD mice. This strategy requires repeated breeding for three generations. B), Average time to reach the platform in the training process of *serpinf1* ^−/−^ 5 × FAD and 5 × FAD mice in MWM test (*n* = 7 mice per group). C–F), Time spent in target quadrant C), platform crossing count D), latency of first time to enter the initial platform E), and representative track and heat map images F) of mice of *serpinf1* ^−/−^ 5 × FAD and 5 × FAD mice in the probe trial of the MWM test. G), Schematic diagram showing the strategy of the AAV vectors engineered to overexpress PEDF in the hippocampus of APP/PS1 mice. H), Representative images of AAV injection sites and infection validation in the hippocampus of APP/PS1 mice. Scale bars: 200 µm (right, magnified view of the left image); 1000 µm (left). I), Average time to reach the platform in the training process of AAV‐*serpinf1* and AAV‐con mice in MWM test (*n* = 5 mice per group). J–M), Time spent in target quadrant J), platform crossing count K), latency of first time to enter the target L) and representative track and heat map images M) of AAV‐*serpinf1* and AAV‐con mice in the probe trial of the MWM test. All data are presented as mean ± S.E.M. Two‐way ANOVA with the Bonferroni's multiple comparison B), I); Comparison by two‐tailed unpaired Student's *t*‐test unless otherwise indicated. ^*^ *p* < 0.05, ^**^ *p* < 0.01; n.s., not significant. See Source data for the statistical details.

To determine whether PEDF deficiency exacerbates progression of the pathological behavioral manifestations of AD and clarify the function of PEDF in AD, we crossed *serpinf1* ^−/−^ mice with 5 × FAD mice (**Figure** **3**A; Figure S4A–G, Supporting Information). 8‐week‐old *serpinf1* ^−/−^ 5 × FAD mice demonstrated impairments to cognitive function in the NOR (Figure S4H–J, Supporting Information), spatial learning memory in the MWM (Figure 3B–F; Figure S4O, Supporting Information), and short‐term working memory in the Y‐maze task (Figure S3K–N, Supporting Information).

Next, to investigate the consequences of overexpressing PEDF in the hippocampus, we infused AAV‐*serpinf1* or AAV‐con viruses into the hippocampal CA1 region of 6‐month‐old APP/PS1 mice (Figure 3G–H; Figure S4P, Supporting Information). In the OFT, no behavioral changes were observed for APP/PS1 mice infected with AAV‐*serpinf1* in the CA1 (Figure S4Q–T, Supporting Information). However, the AAV‐*serpinf1*‐infected mice exhibited improved memory for a novel object in the NOR (Figure S3U–W, Supporting Information) and better spatial learning memory in the MWM (Figure 3I–M; Figure S4b, Supporting Information) and working memory in the Y‐maze task (Figure S4X‐a, Supporting Information). Collectively, these data suggest that reduced expression of PEDF aggravated the behavioral abnormalities in a mouse model of AD, while overexpression of PEDF exerted a protective role.

### 2.4 PEDF Deficiency Coincides With Disrupted Glutamatergic Signaling and Downregulated Astrocytic GLT‐1

**Figure 4:**
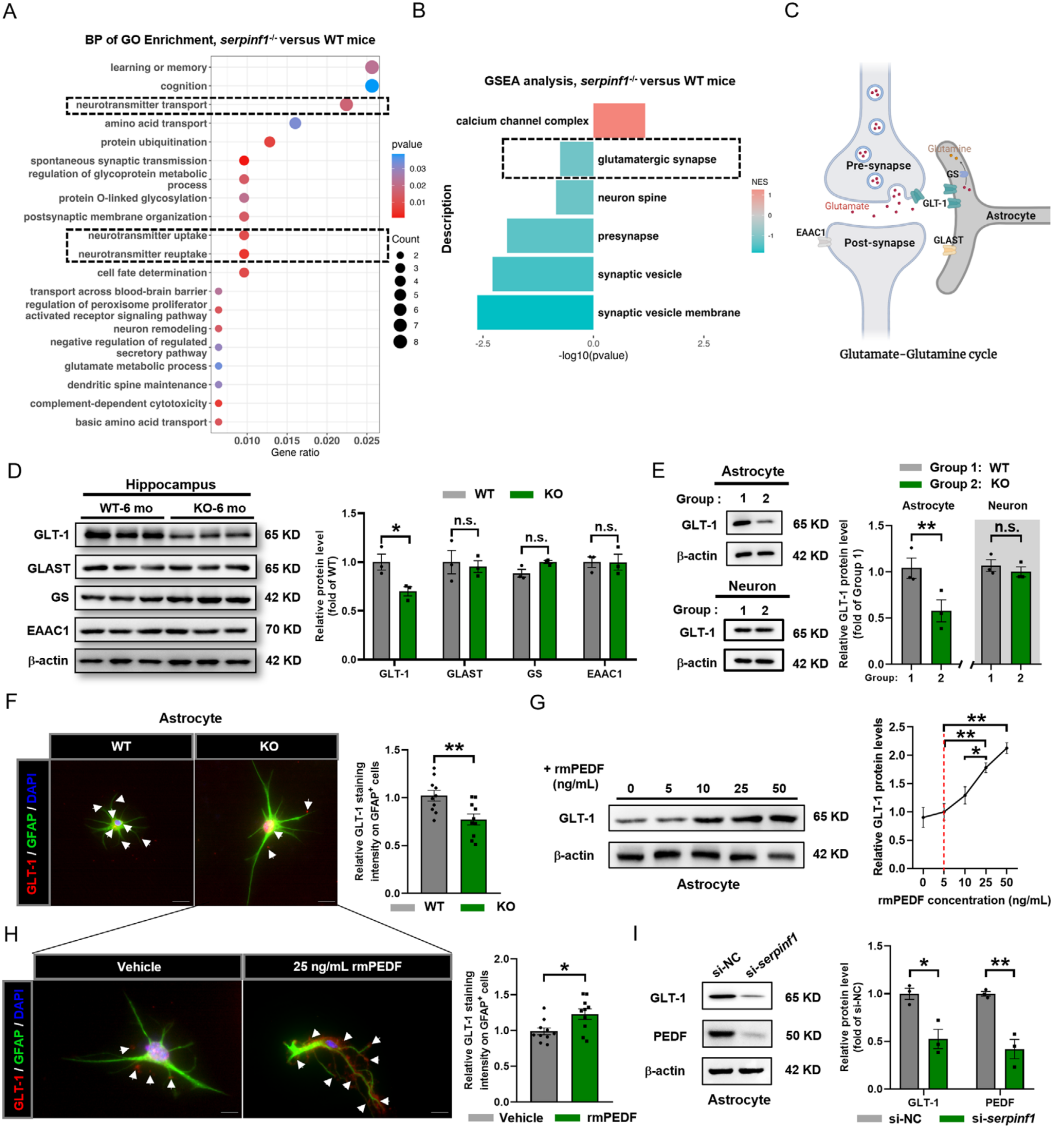
Reduction in astrocytic GLT‐1 expression and glutamatergic signaling under PEDF‐deficient condition. A), The BP category of GO enrichment of DEGs comparing *serpinf1* ^−/−^ mice to WT mice (*n* = 3). Two‐tailed Fisher's exact test and corrected *p* < 0.05 were considered significant. B), Top significantly enriched (normalized enrichment score, NES > 0) and de‐enriched (NES < 0) gene sets revealed by GSEA comparing *serpinf1* ^−/−^ mice to WT mice (*n* = 3). GO‐CC: gene ontology‐cellular component. Discovery Rate (FDR) was calculated to adjust for multiple comparisons, and pathways with FDR < 0.05 were considered statistically significant. C), Overview of the glutamate‐glutamine cycle in the glutamatergic synapses. D), Western blotting analysis of GLT‐1, GLAST, GS, and EAAC1 in the hippocampus of 6‐mo‐old KO and WT mice (left panel). Protein levels were normalized to β‐actin (right panel, *n* = 3 mice per group). E), Western blotting analysis of GLT‐1 in primary cultured astrocytes and neurons of KO and WT P_0_ mice (left panel). Protein levels were normalized to β‐actin (right panel, 3 biological replicates). F), Representative images of GLT‐1 co‐stained with GFAP in primary cultured astrocyte of KO and WT P_0_ mice (left panel); quantification of GLT‐1^+^ intensity on GFAP^+^ cells (right panel). Data point represents primary astrocyte prepared from KO and WT P_0_ mice (*n* = 10 individual cells per group), from three independent experiments. Scale bars: 20 µm. G), Western blotting analysis of GLT‐1 in primary cultured astrocytes at indicated concentration recombinant PEDF (rmPEDF) treatment (left panel). Protein levels were normalized to β‐actin, from three independent experiments (right panel). One‐way ANOVA combined with the Tukey's multiple comparison. H), Representative images of GLT‐1 co‐stained with GFAP in primary cultured astrocyte of KO P_0_ mice at 25 ng mL^−1^ concentration rmPEDF treatment (left panel); quantification of GLT‐1^+^ intensity on GFAP^+^ cells (right panel). Data point represents primary astrocyte prepared from KO P_0_ mice (*n* = 10 individual cells per group), from three independent experiments. Scale bars: 20 µm. I), Western blotting analysis of GLT‐1 and PEDF with small interfering RNAs (si‐*serpinf1* and si‐NC) treatment in primary cultured astrocytes of P_0_ mice (left panel). Protein levels were normalized to β‐actin, from three independent experiments (right panel). All data are presented as mean ± S.E.M. Comparison by two‐tailed unpaired Student's *t*‐test unless otherwise indicated. ^*^ *p* < 0.05, ^**^ *p* < 0.01; n.s., not significant. See Source data for the statistical details.

Next, we investigated the underlying mechanisms by which PEDF regulates AD‐like behavior. Three different categories, BP, cellular compartment (CC), and molecular function (MF), were enriched from DEGs of RNA‐seq analysis between *serpinf1* ^−/−^ and WT mice. In the BP category, synaptic‐related processes, including neurotransmitter transport, neurotransmitter uptake, and reuptake, were enriched in *serpinf1* ^−/−^ mice (**Figure** **4**A; Figure S5A, Supporting Information). Notably, glutamatergic synapses were significantly enriched in *serpinf1* ^−/−^ mice in GSEA analysis of the CC category (Figure 4B).

To confirm the evidence obtained from the GO functional enrichment analysis, we examined the levels of key proteins involved in the glutamate‐glutamine cycle, which is related to neurotransmitter (mainly glutamate) uptake and reuptake function (Figure 4C). WB showed that GLT‐1 was significantly decreased in the hippocampus of 6‐month‐old *serpinf1* ^−/−^ mice compared to WT controls. However, there were no significant changes in the levels of the L‐glutamate‐L‐aspartate transporter (GLAST, also known as EAAT1), glutamine synthetase (GS), or excitatory amino acid carrier 1 (EAAC1, also known as EAAT3) in *serpinf1* ^−/‐^ mice (Figure 4D). The IF staining demonstrated that the fluorescence intensity of GLT‐1 was significantly lower in the hippocampal CA1 region of *serpinf1* ^−/−^ mice than WT controls (Figure S5C, Supporting Information). We also repeated the above experiments in *serpinf1* ^−/−^ and WT mice at 18 months‐of‐age (Figure S5B–D, Supporting Information).

GLT‐1 is mainly expressed in astrocytes, with a small fraction also expressed at the axon terminals of neurons (Figure 4C). WB showed that GLT‐1 was significantly decreased in cultured astrocytes, but not neurons (Figure 4E). Additionally, IF staining demonstrated that GLT‐1‐positive area was significantly smaller in *serpinf1* ^−/−^ astrocytes compared to WT controls (Figure 4F).

When treated with recombinant mouse‐derived PEDF protein (rmPEDF), WB showed that GLT‐1 expression increased in a concentration‐dependent manner above 25 ng mL^−1^ (physiological concentration in mice: 5 ng mL^−1^, 1/1000 of that in humans^[^ ^34^ ^]^) in both primary astrocytes (Figure 4G; Figure S5E, Supporting Information) or C6 cell line (Figure S5H, Supporting Information). IF staining confirmed that the GFAP‐labeled astrocytes from *serpinf1* ^−/−^ mice expressed higher levels of GLT‐1 after treatment with rmPEDF (Figure 4H).

Furthermore, WB analysis confirmed overexpression of GLT‐1 in primary astrocytes (Figure S5F,G, Supporting Information) or C6 cell line (Figure S5I, Supporting Information) transfected with a PEDF‐overexpressing plasmid with pcDNA3.1^+^ as vector. Furthermore, WB and IF staining analysis revealed a significant decrease in GLT‐1 expression in primary astrocytes transfected with a small interfering RNA for PEDF (si‐*serpinf1*) (Figure 4I; Figure S5J,K, Supporting Information). Overall, these results indicate that PEDF regulates the expression of astrocytic GLT‐1 in the hippocampus.

### 2.5 PEDF Deficiency Disrupts Glutamate Homeostasis and Synaptic Transmission

**Figure 5:**
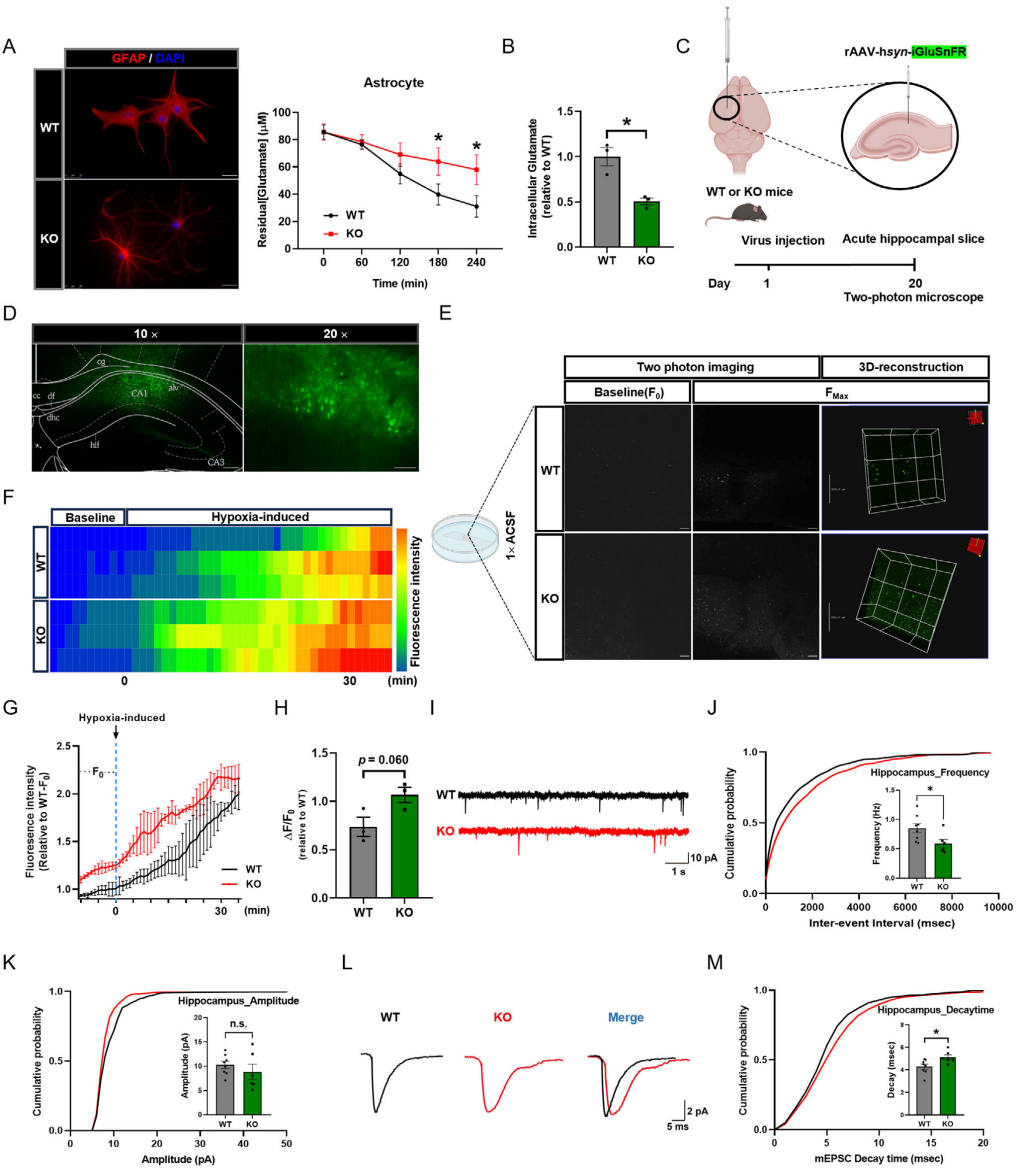
PEDF deficiency disrupts astrocytic glutamate uptake ability and glutamate homeostasis in mice. A), Representative confocal micrographs of the morphology of primary astrocyte prepared from KO and WT P_0_ mice (left panel). Astrocytes incubated in an uptake buffer containing 100 µm unlabeled glutamate for extracellular glutamate assay: Extracellular glutamate concentrations were determined in samples from the uptake buffer collected at the indicated time points (right panel). Data were verified in three independent experiments. Scale bars: 25 µm. B), Intracellular glutamate concentration after the extracellular glutamate assay, from three independent experiments. C,D), Schematic of the AAV vectors engineered to express the glutamate sensor (iGluSnFR) under a *syn* promoter C) and representative images of the injection sites in the hippocampus D). Scale bars: 50 µm (right, magnified view of the left image); 500 µm (left). E), Representative 2D and 3D images of baseline and maximum glutamate signaling in the hippocampal slices of KO and WT mice. Scale bars: 20 µm (2D); 200 µm (3D). F,G), Representative heatmap of average iGluSnFR intensity during the time course F) and quantification of the average fluorescence intensity G) (*n* = 3 mice per group). H), Quantification of pre‐ and post‐hypoxia stimulated glutamate changes by the ratio of the difference between the baseline and maximum level of the glutamate signal to the baseline (*n* = 3 mice per group). I), Representative traces of mEPSCs recorded from WT and KO mice. J,K), Cumulative distribution and mean mEPSC frequency j) and amplitudes k) (*n* = 6–8 cells from 3 individual mice). L), Representative traces of mean individual mEPSCs from WT and KO mice. M), Cumulative distribution and mean value of mEPSC decay time (*n* = 8 WT group; *n* = 6 KO group, from 3 individual mice per group). All data are presented as mean ± S.E.M. Two‐way ANOVA with the Bonferroni's multiple comparison A,G); Comparison by two‐tailed unpaired Student's *t*‐test unless otherwise indicated. ^*^ *p* < 0.05, ^****^ *p* < 0.0001; n.s., not significant. See Source data for the statistical details.

The glutamate concentration was reduced by almost 50% after 4 h in the WT astrocytes, while the glutamate‐uptake ability was significantly impaired in *serpinf1* ^−/−^ astrocytes (**Figure** **5**A). In addition, *serpinf1* ^−/−^ astrocytes had significantly lower intracellular glutamate concentrations than WT astrocytes, indicating KO‐astrocytes had lower glutamate‐uptake ability (Figure 5B). Moreover, transfection with the PEDF‐overexpressing adenovirus improved the glutamate‐uptake ability of C6, but did not alter glutamate‐release (Figure S6A–E, Supporting Information).

Next, AAV‐iGluSnFR virus was infused into the hippocampus of 6‐month‐old *serpinf1* ^−/−^ and WT mice, and acute hippocampal slices were obtained at 20 days after infection and observed by two‐photon microscopy (Figure 5C–E). Analysis of glutamate‐signaling revealed *serpinf1* ^−/−^ mice exhibited higher baseline and maximum glutamate signaling after hypoxic stimulation than the WT mice (Figure 5E–G). Although the *serpinf1* ^−/−^ mice demonstrated higher glutamate release than WT control mice, the difference was not significant (Figure 5H). Furthermore, we obtained consistent results in the offspring of 8‐week‐old 5 × FAD mice crossed with *serpinf1* ^−/−^ 5 × FAD mice (Figure S6F–I, Supporting Information).

*Serpinf1* ^−/−^ mice exhibited a significantly lower frequency and amplitude of miniature excitatory postsynaptic currents (mEPSCs) compared to WT controls (Figure 5I–K). Additionally, the decay time for single mEPSCs was elevated in *serpinf1* ^−/−^ mice, indicating longer glutamate clearance in synapses (Figure 5L,M). These results indicate reduced expression of PEDF disrupted basal glutamatergic synaptic transmission, a phenomenon in accordance with the alterations observed at the level of glutamatergic synaptic proteins, including vesicular glutamate transporter 1 (VGlut1) (Figure S6J, Supporting Information). Moreover, GABAergic synapses did not appear to be affected in *serpinf1* ^−/−^ mice (Figure S6K, Supporting Information). Furthermore, *serpinf1* ^−/−^ mice exhibited abnormal activation of astrocytes at 6 months‐of‐age and the levels of activation progressively deteriorated with age, whereas no significant changes in either microglial number or activity status were observed in *serpinf1* ^−/−^ mice (Figure S6L–N, Supporting Information).

Notably, impaired synaptic glutamate clearance has been previously implicated in vulnerability to epileptic seizures.^[^ ^35^ ^]^ As anticipated, intraperitoneal administration of a single subthreshold dose of 2 mg kg^−1^ kainic acid did not cause any seizure‐related response in WT control mice. However, most of the KO mice developed clonic seizures as early as 30–40 min after injection (Figure S6O, Supporting Information). Thus, these data confirm that the loss of PEDF leads to functional alterations in the synapses of the hippocampus.

### 2.6 Loss of PEDF Impairs Neuronal Morphology and Synaptic Plasticity

**Figure 6:**
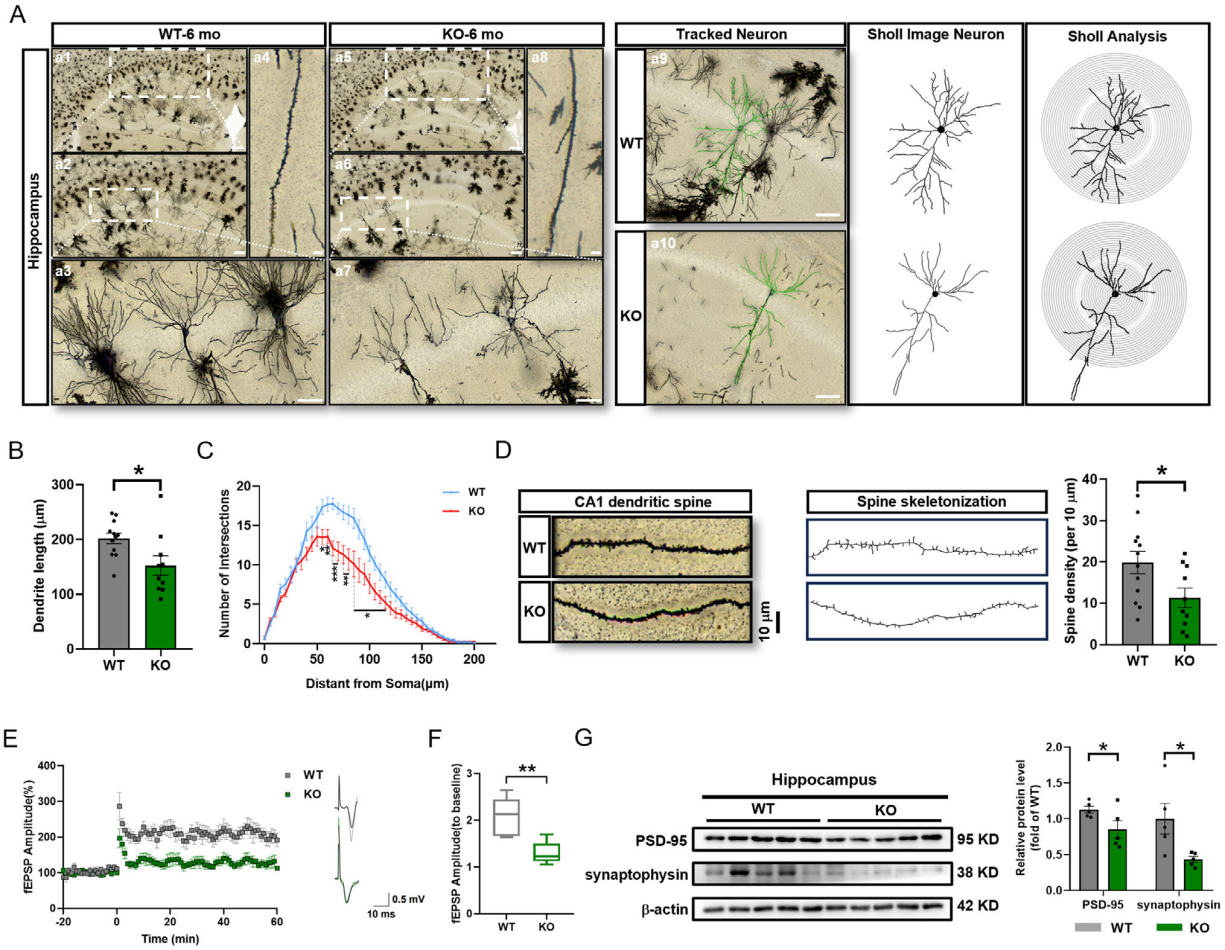
PEDF deficiency impairs dendritic spine morphology and LTP formation. A), Representative confocal micrographs of the morphology of pyramidal neurons in the hippocampus of 6‐mo‐old WT and KO mice (left panel). 3D neuron tracking reconstruction and Sholl analysis images (right panel). Scale bar: 500 µm (a1, a5), 200 µm (a2, a6), 50 µm (a3, a7, Track Neuron), 20 µm (a4, a8). B,C), Total cumulative lengths of basal dendritic processes neuron (B) and Sholl analysis of dendritic arbors of pyramidal neurons C) in the hippocampus (*n* = 10–12 cells from 3 individual mice per group). D), Representative confocal micrographs and spines‐skeletonized images of dendritic spines of pyramidal neurons in the hippocampus of WT and KO mice (left panel). Scale bar: 10 µm. Quantitative analysis of dendritic spine density (*n* = 10–12 cells from 3 individual mice per group) (right panel). One‐way ANOVA with Fisher's LSD test. E), A 100‐Hz high‐frequency stimulation (HFS)‐induced LTP was recorded in the hippocampus slices from KO and WT mice (left panel). Representative fEPSP waveform recordings (right panel) (*n* = 6 slices from 3 mice in WT group, *n* = 5 slices from 3 mice in KO group). F), Ratio of mean to baseline fEPSP amplitude in the last 10 min after high‐frequency stimulation (*n* = 3 mice per group). G), Western blotting analysis of PSD‐95 and synaptophysin in the hippocampus of WT and KO mice (left panel). Protein levels were normalized to β‐actin (right panel, *n* = 5 mice per group). All data are presented as mean ± S.E.M. Two‐way ANOVA with the Bonferroni's multiple comparison C); Comparison by two‐tailed unpaired Student's *t*‐test unless otherwise indicated. ^*^ *p* < 0.05, ^**^ *p* < 0.01, ^***^ *p* < 0.001; n.s., not significant. See Source data for the statistical details.

Neurons adjust their dendritic growth and branching to compensate for changes in synaptic transmission, and these processes are termed structural homeostasis.^[^ ^36^ ^]^ Previous reports showed that abnormally high levels of extracellular glutamate cause dendritic atrophy and spine loss.^[^ ^37^ ^]^ Sholl analysis after Golgi staining confirmed reduced dendrite complexity and dendritic spine density in CA1 neurons from *serpinf1* ^−/−^ mice at 6 months‐of‐age (**Figure** **6**A–D), and more significant reductions at 18 months‐of‐age (Figure S7A–D, Supporting Information).

Long‐term potentiation (LTP) is considered to be the neurological basis of learning memory.^[^ ^38^ ^]^ We therefore recorded the Schaffer collaterals (SC) for CA1 pathway LTP in *serpinf1* ^−/−^ and WT mice. Based on a 30 min baseline recording of field excitatory postsynaptic potential (fEPSP) compared with the first 10 min and last 10 min after high‐frequency stimulation (HFS), 9‐month‐old WT mice induced normal LTP. In contrast, *serpinf1* ^−/−^ mice exhibited an impairment in HFS‐induced LTP (Figure 6E‐F). Previous studies have shown that LTP in the SC‐CA1 of the hippocampus is dependent on N‐methyl‐D‐aspartate receptor (NMDAR)‐mediated excitatory postsynaptic currents (EPSCs).^[^ ^39^ ^]^ Accordingly, whole‐cell recording of CA1 pyramidal neurons revealed significantly smaller amplitudes of NMDAR‐mediated EPSC in the neurons of 6‐month‐old *serpinf1* ^−/−^ mice than WT controls (Figure S7E,F, Supporting Information). WB confirmed reduced levels of synaptophysin and postsynaptic density protein 95 (PSD‐95) in the hippocampus of *serpinf1* ^−/−^ mice. (Figure 6G).

Reduced astrocytic GLT‐1 levels may elevate the concentration of glutamate at synapses and subsequently trigger excitotoxic cell death via a NMDAR–mediated increase in intracellular Ca^2+^.^[^ ^40^ ^]^ Nissl staining did not reveal any significant differences in the hippocampal CA1 region of *serpinf1* ^−/−^ mice at 6, 9, and 12 months‐of‐age. However, neuronal loss was significantly greater in *serpinf1* ^−/−^ mice at 15 months‐of‐age compared to WT controls (Figure S7G, Supporting Information) and by 18 months‐of‐age, *serpinf1* ^−/−^ exhibited significant neuronal loss in other regions of the hippocampus, including CA3 and DG (Figure S7H, Supporting Information). Furthermore, s*erpinf1* ^−/−^ 5 × FAD mice also exhibited significantly greater neuronal loss than 5 × FAD mice (Figure S8A, Supporting Information). On the contrary, infusion of AAV‐*serpinf1* into the hippocampus CA1 of APP/PS1 mice at 6 months‐of‐age attenuated neuronal loss (Figure S8B, Supporting Information).

Changes in glutamate transporter expression have been identified to occur at an early stage of AD, which indicates that dysfunctional glutamate transport might be an early event in the pathology of the disease.^[^ ^41^ ^]^ Interestingly, glutamate and amyloid β have been shown to affect each other.^[^ ^42^ ^]^ Apart from affecting amyloid β production, there is evidence that interactions between glutamate and amyloid β influence tau phosphorylation.^[^ ^43^ ^]^ IF staining revealed that neither amyloid β production nor tau phosphorylation was significantly altered in *serpinf1* ^−/−^ mice at 6 months‐of‐age (Figure S8C–E, Supporting Information). However, at 18 months‐of‐age, both soluble amyloid β and tau phosphorylation were significantly increased in the hippocampus of *serpinf1* ^−/−^ mice compared to WT controls (Figure S8F–H, Supporting Information). Notably, AAV‐*serpinf1* treatment led to a marked reduction in amyloid β deposition in the hippocampal CA1 region of APP/PS1 mice, as evidenced by Thioflavin‐T (Th T) staining (Figure S8I). Consistently, it also significantly attenuated phosphorylated tau level (Figure S9A–C, Supporting Information). Taken together, these data indicate depletion of PEDF increases the concentration of glutamate at synapses in the early stage of AD by reducing the expression of GLT‐1 astrocytes, and these changes significantly contribute to the age‐related progression of AD pathology, including impaired synaptic plasticity, neuronal loss, amyloid β production and tau phosphorylation.

### 2.7 Reduction of PEDF Facilitates PKCα Phosphorylation‐Activated Ubiquitin‐Proteasome‐Dependent Degradation of GLT‐1

**Figure 7:**
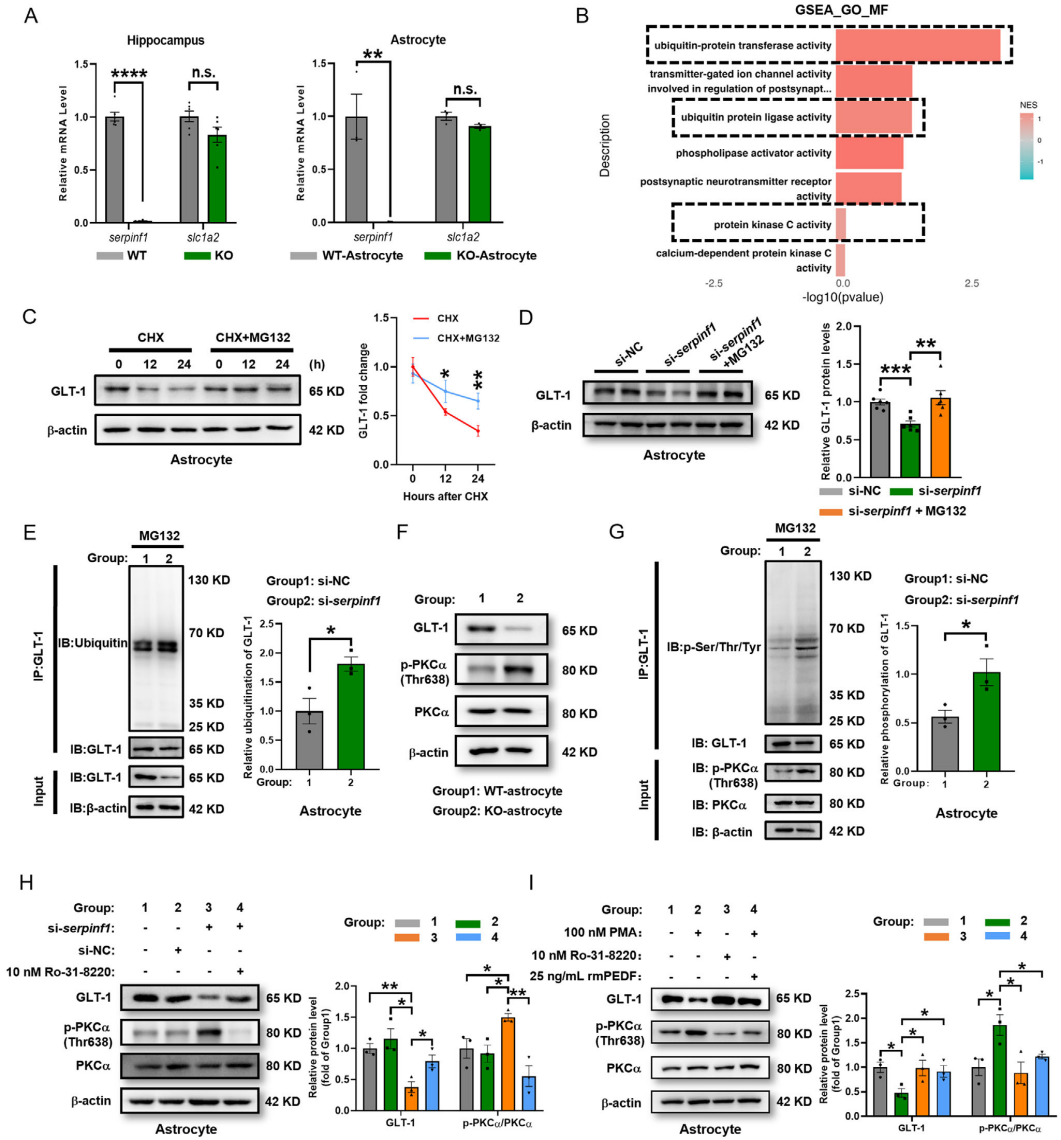
PEDF deficiency drives abnormal PKCα phosphorylation‐activated, leading to the increasing ubiquitin‐proteasome‐dependent degradation of GLT‐1. A), Gene expression of *serpinf1* and *slc1a2* mRNA in the hippocampus (left panel) and primary astrocyte (right panel) of WT and KO mice (*n* = 6 mice per group; 3 biological replicates). B), Top enriched (NES > 0) gene sets revealed by GSEA. GO‐MF: gene ontology‐molecular function. C), Western blotting analysis and quantification of GLT‐1 in primary cultured astrocytes incubated in 100 µg mL^−1^ CHX with or without MG132 treatment at the indicated time points. D), Western blotting analysis and quantification of GLT‐1 in control (si‐NC), PEDF‐silenced (si‐*serpinf1*), or PEDF‐silenced with MG132 treatment primary cultured astrocytes. E), Astrocytes were transfected with si‐NC or si‐*serpinf1* in the presence of MG132. GLT‐1 was immunoprecipitated (IP). Western blotting analysis and quantification of ubiquitinated proteins or GLT‐1. F), Representative western blotting analysis and quantification of GLT‐1, PKCα, and p‐PKCα in primary cultured astrocytes of WT and KO P_0_ mice. G), Astrocytes were transfected with si‐NC or si‐*serpinf1* in the presence of MG132. GLT‐1 was IP. Western blotting analysis and quantification of phosphorylated proteins and GLT‐1 (left panel). Protein levels were normalized to β‐actin, from three independent experiments (right panel). H), Representative western blotting analysis and quantification of GLT‐1, PKCα, and p‐PKCα in astrocytes treated with si‐NC, si‐*serpinf1*, Ro‐31‐8220 (10 nm), or both for 24 h, as indicated. I), Representative western blotting analysis and quantification of GLT‐1, PKCα, and p‐PKCα in astrocytes treated with PMA (100 nm), Ro‐31‐8220 (10 nm), rmPEDF (25 ng mL^−1^), or both for 24 h, as indicated. Protein levels were normalized to β‐actin, from three independent experiments. All data are presented as mean ± S.E.M. Two‐way ANOVA with the Bonferroni's multiple comparison C,H,I); Comparison by two‐tailed unpaired Student's *t*‐test unless otherwise indicated. ^*^ *p* < 0.05, ^**^ *p* < 0.01, ^***^ *p* < 0.001, ^****^ *p* < 0.0001; n.s., not significant. See Source data for the statistical details.

To investigate the mechanisms by which deficiency of PEDF reduces astrocytic expression of GLT‐1, we quantified *Slc1a2* mRNA (gene name of GLT‐1) in hippocampal tissues and primary astrocytes isolated from *serpinf1* ^−/−^ and WT mice by RT‐qPCR. In line with our previous RNA‐seq analysis of hippocampal tissues from *serpinf1* ^−/−^ and WT mice, PEDF deficiency did not transcriptionally modulate astrocytic GLT‐1 (**Figure** **7**A). Notably, the GSEA analysis of the CC category from our RNA‐seq analysis indicated that the ubiquitin‐dependent proteasome degradation pathway is significantly activated in the hippocampus of *serpinf1* ^−/−^ mice (Figure 7B; Figure S10A, Supporting Information).

GLT‐1 is a relatively stable protein.^[^ ^44^ ^]^ However, GLT‐1 can be ubiquitinated and degraded by the proteasome,^[^ ^45^ ^]^ and treating astrocytes with the proteasomal inhibitor MG132 elevated the level of GLT‐1. Aberrant ubiquitination of GLT‐1 was reported to reduce both GLT‐1 protein expression and glutamate uptake in astrocytes.^[^ ^46^ ^]^ Accordingly, we inhibited protein using cycloheximide (CHX) to examine the half‐life of GLT‐1 in C6 cells. The protein levels of GLT‐1 decreased significantly as the duration of CHX treatment increased. However, these effects were partially rescued by the addition of MG132, by which the half‐life period of GLT‐1 was estimated to be around 12 h (Figure S10B, Supporting Information). Similar trends were observed in primary astrocytes treated with CHX for 12 and 24 h (Figure 7C). The results showed that GLT‐1 protein levels were lower in si‐*serpinf1* cells compared to si‐NC cells, however, the reduction in GLT‐1 in the si‐*serpinf1* cells was significantly alleviated by the addition of MG132, suggesting that deficiency of PEDF enhances proteasomal degradation of GLT‐1 (Figure 7D). Furthermore, the co‐immunoprecipitation (Co‐IP) analysis showed a significant increase in ubiquitin‐binding GLT‐1 following knockdown of PEDF expression on primary astrocytes (Figure 7E). Conversely, in primary astrocytes pretreated with CHX for 24 h, we observed significantly elevated protein GLT‐1 expression after treatment with rmPEDF, which suggests that the presence of PEDF attenuated degradation of GLT‐1 (Figure S10C, Supporting Information). In parallel, co‐IP assays demonstrated that treatment of primary astrocytes with rmPEDF significantly reduced ubiquitin‐binding GLT‐1 (Figure S10D, Supporting Information).

Ubiquitination of GLT‐1 is strongly associated with activation of protein kinase C alpha (PKCα).^[^ ^47^ ^]^ Notably, in our previous RNA‐seq analysis, GSEA analysis of the MF category revealed increased activation of PKC in *serpinf1* ^−/−^ mic (Figure 7B). Thus, to examine the interaction between GLT‐1 and phosphorylation of PKCα, we performed WB of phosphorylated PKCα (Thr638). PKCα was significantly activated in primary P_0_ astrocytes extracted from the hippocampus of *serpinf1* ^−/−^ mice compared with WT controls (Figure 7F). Subsequently, cultured astrocytes were collected, immunoprecipitated with GLT‐1, and blotted with pan‐serine/threonine/tyrosine phosphorylation and GLT‐1 antibodies. Co‐IP assays showed that the levels of phosphorylated GLT‐1 were higher in cells transfected with si‐*serpinf1*, with the input group showing a significant increase in phosphorylated PKCα (Thr638), which suggests that activation of PKCα may lead to phosphorylation of GLT‐1 (Figure 7G). Additionally, inhibition of PKCα activation in cells transfected with si‐*serpinf1* using the PKC inhibitor α Ro‐31‐8220 increased the expression of GLT‐1 (Figure 7H). As further reverse validation, treatment with phorbol 12‐myristate 13‐acetate (PMA), an agonist of PKCα, confirmed that activation of PKCα significantly decreased the expression of GLT‐1 in astrocytes. However, treatment with rmPEDF significantly alleviated the decrease in GLT‐1 expression caused by activation of PKCα using PMA (Figure 7I). IF staining showed treatment with PMA reduced GLT‐1 expression in astrocytes, and this effect could be rescued by the addition of rmPEDF (Figure S10E, Supporting Information). Interestingly, PKCα expression was not significantly different in the hippocampus of *serpinf1* ^−/−^ mice and WT controls. However, the levels of phosphorylated PKCα were significantly higher in *serpinf1* ^−/‐^ mice, suggesting that a lack of PEDF may endogenously activate PKCα (Figure S10F, Supporting Information).

To undertake a preliminary investigation of the pathways by which PEDF inhibits the activation of PKCα, we investigated the structure of PEDF. Previous studies have reported that the neuroprotective function of PEDF is derived from a 44 amino acid‐containing peptide (Val78‐Thr121)^[^ ^48^ ^]^ that acts as a ligand and binds to the PEDF receptor (PEDF‐R, also known as Adipose Triglyceride Lipase, ATGL; encoded by the *PNPLA2* gene) (Figure S10G, Supporting Information). Therefore, we first investigated the basal levels of PEDF‐R in the mouse brain and found that PEDF‐R is highly expressed in the hippocampus (Figure S10H, Supporting Information). PEDF‐R, as an defined (ATGL) that exhibits esterase function, degrades triglycerides to produce diacylglycerol (DAG), which can increase PKC activity.^[^ ^49^ ^]^ We did not observe any significant differences in DAG expression in primary astrocytes from *serpinf1* ^−/−^ and WT mice (Figure S10I, Supporting Information). Thus, we knocked down PEDF‐R in C6 cells using a small interfering RNA (si‐*pnpla2*), which increased the phosphorylation of PKCα, and inhibition of PKCα phosphorylation by rmPEDF was blocked (Figure S10J–L, Supporting Information). Notably, PEDF‐R can exert calcium‐independent phospholipase A_2_ (iPLA_2_) activity upon binding to PEDF, which stimulates the release of docosahexaenoic acid (DHA) from cell membrane phospholipids, where DHA has been reported to inhibit the activity of PKCα.^[^ ^50^ ^]^ In agreement with these reports, knockdown of PEDF‐R using si‐*pnpla2* in C6 cells resulted in significantly lower levels of DHA (Figure S10M, Supporting Information). These data suggest that PEDF deficiency may promote phosphorylation of PKCα, which increases ubiquitin proteasomal degradation of GLT‐1 by blocking the PEDF‐R/iPLA_2_/DHA pathway.

### 2.8 Upregulation of GLT‐1 Rescues Cognitive Impairment in *serpinf1^−/−^* Mice

If decreased GLT‐1 expression is a major cause of cognitive decline in *serpinf1* ^−/−^ mice, we hypothesized that increasing GLT‐1 expression may improve spatial learning and short‐term working memory. We injected saline or ceftriaxone (CEF), a β‐lactam antibiotic, which has been reported to exert neuroprotective effects by upregulating the transcription of GLT‐1^51^, into 15‐month‐old *serpinf1* ^−/−^ mice for 5 days and carried out cognitive memory behavior tests (Figure S11A, Supporting Information). *Serpinf1* ^−/−^ mice that received CEF spent more time interacting with the novel arm than the saline group in the Y‐maze test, indicating CEF improved their short‐term working memory, although the effect was not significant (Figure S11B–E, Supporting Information). CEF‐injected *serpinf1* ^−/−^ mice showed a better learning curve during the escape latency period and improved memory for the platform position in the test phase compared to saline‐injected *serpinf1* ^−/−^ mice in the MWM test (Figure S11F–K, Supporting Information) and improved contextual memory in an injection‐based fear conditioning test (Figure S11L–N, Supporting Information). Injection of CEF significantly increased the mRNA and protein levels of GLT‐1 in the hippocampus tissues. However, the protein level GLT‐1 in CEF‐injected *serpinf1* ^−/−^ mice was still significantly lower compared to saline‐injected WT mice, which suggested that CEF may have improved the behavioral performance of *serpinf1* ^−/−^ mice through other effects (Figure S11O,P, Supporting Information). Nissl staining showed a trend towards a higher number of normal neurons in *serpinf1* ^−/−^ treated with CEF. However, this effect was not significant compared with the *serpinf1* ^−/−^ saline mice (Figure S11Q, Supporting Information).

**Figure 8:**
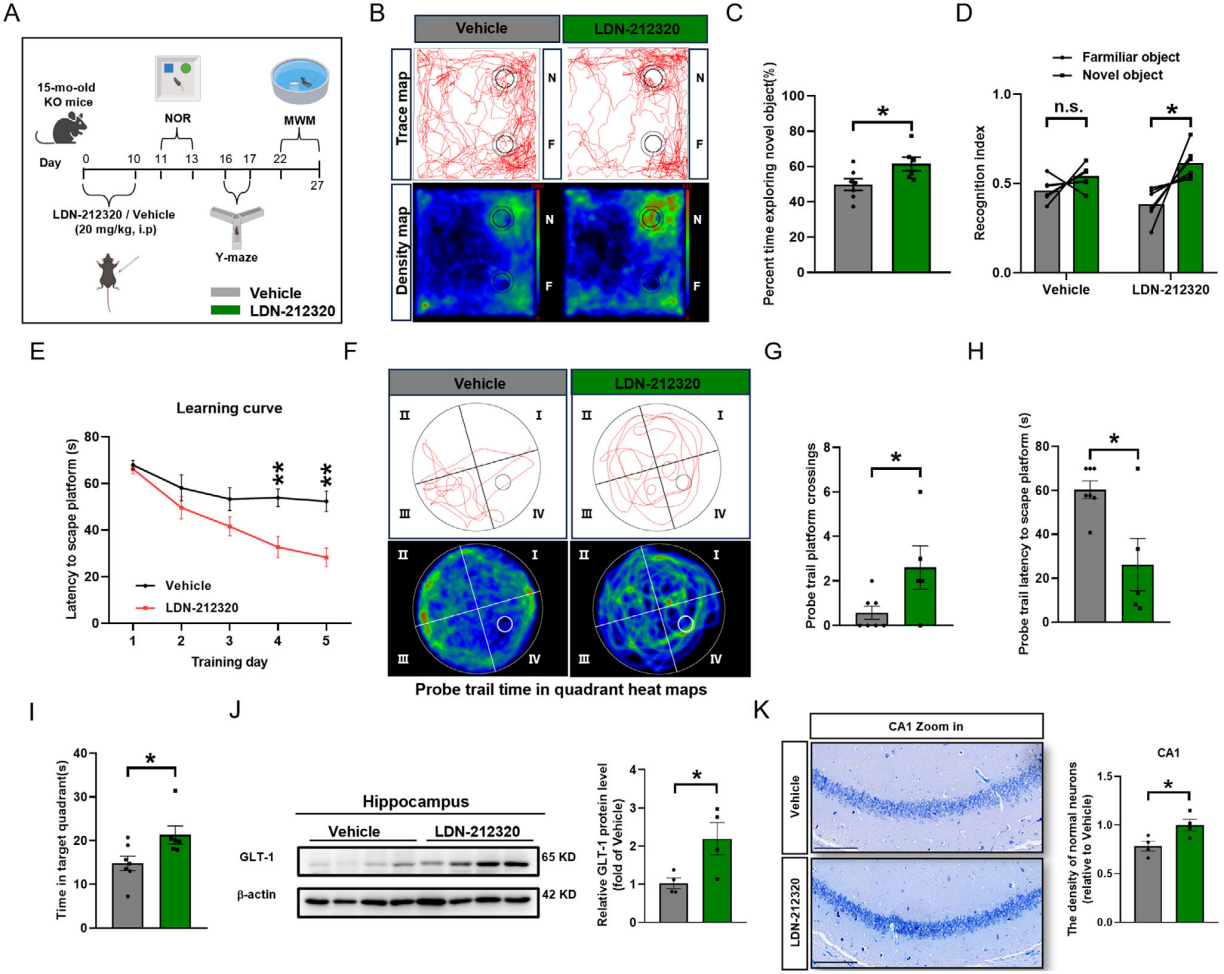
Enhancing GLT‐1 expression by LDN‐212320 rescues learning and memory impairment in PEDF deficient mice. A), Schematic diagram showing the LDN‐212320 treatment strategy in KO mice. B), Representative track and heat map images by KO mice treated with vehicle and LDN‐212320 in the NOR test. C) and D), Quantification of percentage time spent in the novel and familiar object C), and discrimination index D) during the test phase (*n* = 7 mice of vehicle group, *n* = 6 mice of LDN‐212320 group). E), Latency to reach the hidden platform during spatial training in the MWM. One‐way ANOVA with Fisher's LSD test. F–I), Representative track and heat map images of mice F), platform crossing count G), latency of first time to enter the target H), and time spent in target quadrant I) in the probe trial of the MWM test. J), Western blotting analysis and quantification of GLT‐1 in the hippocampus of KO mice treated with vehicle and LDN‐212320 (*n* = 4 mice per group). K), Representative images of Nissl staining of the hippocampal CA1 of KO mice treated with vehicle and LDN‐212320 (left panel); quantification of the density of normal neurons (right panel) (*n* = 4 mice per group). Scale bar: 100 µm. All data are presented as mean ± S.E.M. Two‐tailed paired Student's *t*‐test D); Two‐way ANOVA with Bonferroni's multiple comparison E); Comparison by two‐tailed unpaired Student's *t*‐test unless otherwise indicated. ^*^ *p* < 0.05, ^**^ *p* < 0.01; n.s., not significant. See Source data for the statistical details.

Due to the limited effects of CEF treatment and difficulty of exclude the influence of other mechanisms of action of CEF as an antibiotic, we employed LDN‐212320 (also known as OSU‐212320), a small‐molecule agonist that has been reported to extend the lifespan of mice in a model of amyotrophic lateral sclerosis (ALS), to exert a neuroprotective function by increasing translation of GLT‐1.^[^ ^52^ ^]^ We injected *serpinf1* ^−/−^ mice with 20 mg/kg LDN‐212320 or vehicle for 10 consecutive days and assessed cognitive memory behavior (**Figure** **8**A). In the NOR, LDN‐212320‐treated mice exhibited a significantly longer exploration time for the new object and better recognition ability, suggesting LDN‐212320 improved the cognitive function of *serpinf1* ^−/−^ mice (Figure 8B–D). Similarly, LDN‐212320 significantly improved discrimination between the old and new arm in the Y‐maze test by *serpinf1* ^−/−^ mice (Figure S12A‐D). In the MWM task, LDN‐212320‐treated *serpinf1* ^−/−^ mice exhibited a significantly shorter escape latency during the orientation navigation phase from day 2 until day 5 (Figure 8E) and a significantly better exploration strategy for the platform during the spatial exploration phase (Figure 8F–I; Figure S12E, Supporting Information). In confirmation of the rescue effect, WB analysis showed that LDN‐212320 significantly increased GLT‐1 protein expression in the hippocampus (Figure 8J). Finally, Nissl staining of the hippocampus showed a significant reduction in neuronal loss in *serpinf1* ^−/−^ mice in response to LDN‐212320 treatment (Figure 8K). These data demonstrate that LDN‐212320 effectively increased GLT‐1 protein expression through translation and attenuated the development of neurodegenerative lesions in *serpinf1* ^−/−^ mice.

## 3 Discussion

This study identified a novel role for PEDF in the maintenance of glutamate homeostasis and cognitive function. Our data show that a deficiency of PEDF leads to aberrant extracellular glutamate concentration, synaptic plasticity, and altered dendritic spine morphology as a result of glutamate excitotoxicity due to reduced expression of GLT‐1 in astrocytes, and has long‐lasting consequences on cognitive behavior in the mouse.

To address the controversy surrounding whether PEDF expression is altered in AD, our comprehensive analysis of multiple RNA‐seq databases provided strong evidence for decreased expression of PEDF in the hippocampus of patients with AD. Furthermore, we verified the reduction in PEDF levels through a combination of blood tests and scale assessments in a relatively large cohort of patients with AD and individuals without dementia. Through the analysis of a previous proteomics report, it was found that the level of PEDF in CSF was also significantly reduced in the AD population. Thus, we speculate that the levels of PEDF are systemically decreased in AD, in line with the expectation that PEDF levels in peripheral blood may be indicative of PEDF levels in the central nervous system (CNS). Notably, our previous study provided conclusive evidence that PEDF levels decline with age.^[^ ^32^ ^]^ Our data also suggested there is a close correlation between reduced PEDF levels and cognitive deficits, which could potentially represent a theoretical molecular basis for aging as a risk factor for AD. Thus, assessment of PEDF may hold potential a valuable diagnostic indicator for the onset and progression of age‐related diseases. Future studies will require larger, pathologically confirmed AD cohorts combined with paired CSF/blood to be realized.

There is prior evidence for impaired glutamate homeostasis in AD.^[^ ^53^ ^]^ Glutamate homeostasis plays essential roles in dendritic spine morphology, the postsynaptic proteins that regulate dendritic spine morphology, glutamate signaling, and the physiology of glutamate synaptic.^[^ ^6^ ^]^ Our study provides the first demonstration that PEDF plays an indispensable physiological role in glutamate homeostasis by modulating astrocytic GLT‐1. Glutamate is a potent excitotoxin that invokes rapid‐ and delayed‐type neurotoxicity.^[^ ^54^ ^]^ The synapse loss observed in our PEDF‐deficient mice seems to represent a form of delayed neuronal death. Moreover, loss of PEDF promoted vulnerability to epileptic seizures in response to sub‐threshold doses of kainic acid, which may be due to increased accumulation of glutamate in the hippocampus. It is hypothesized that a deficiency of PEDF in the early stage of AD may lead to further continuous deterioration during the progression of the disease, which implies that PEDF is an essential factor in limiting AD progression. Undeniably, there may be other systemic effects of the PEDF‐deficient model,^[^ ^55^, ^56^ ^]^ while our study focused on verifying the causal role of the PEDF‐GLT‐1 axis in ad‐related neurodegeneration. Limitations do not detract from the core conclusion that our study reveals a key mechanism of the PEDF‐GLT‐1 pathway in AD.

The focus on the male mouse model in our study is primarily based on the fact that estrogen has been shown to significantly downregulate PEDF expression,^[^ ^57^, ^58^, ^59^ ^]^ and using males avoids the variability induced by the estrous cycle. Specifically, female AD mice exhibited lower survival rates compared to males, which could introduce confounding variables and complicate data interpretation.^[^ ^60^, ^61^ ^]^ Additionally, hormonal fluctuations in females could have added further variability, making it difficult to maintain experimental consistency. These factors led us to focus on male mice. We acknowledge that the exclusion of female subjects is a limitation of the present study. Given the growing recognition of sex as a biological variable in neurodegenerative research, future investigations are warranted to evaluate potential sex‐specific responses to PEDF treatment. Incorporating both male and female models will be essential to fully elucidate the therapeutic potential and translational relevance of PEDF in AD.

Ubiquitination is implicated in endocytosis and degradation of numerous membrane proteins, including other neurotransmitter transporters such as the dopamine transporter.^[^ ^62^ ^]^ Endocytosis of GLT‐1 depends on its ubiquitination, which is triggered by activated PKCα.^[^ ^63^ ^]^ GLT‐1 can be phosphorylated at multiple serine, threonine, and tyrosine residues,^[^ ^64^ ^]^ thus we used a pan‐serine/threonine/tyrosine antibody. Ubiquitination is also necessary for the trafficking and degradation of GLT‐1.^[^ ^65^ ^]^ Our results suggest that deficiency of PEDF promotes PKCα activation‐dependent ubiquitination of GLT‐1. Activation of PKCα is strongly implicated in the progression of AD, mutations in PKCα significantly increase the risk of AD, and a slight increase in PKCα activity sufficient to lead to the development of an AD phenotype in mice.^[^ ^66^ ^]^ Thus, our data indicate that PEDF may act as a novel endogenous inhibitor of PKCα activity. Furthermore, in addition to mutations in PKCα, deficiency of PEDF may be a key mechanism that leads to endogenous activation of PKCα. However, further research is required to determine whether the PEDF deficiency‐mediated activation of PKCα, which we observed in astrocytes also occurs in other cell types.

Glutamate‐receptor antagonists have not been a very successful strategy in humans because they affect normal synaptic signaling and lead to adverse side effects by blocking the physiological functions of the receptor. Memantine is a relatively safe drug with few side effects, but it exerts inadequate clinical effects on cognition, global functioning, and activities of daily living.^[^ ^67^ ^]^ In neurodegenerative disorders, an insufficient level of GLT‐1 is considered to be the leading cause of excitotoxicity.^[^ ^68^ ^]^ Clinical studies and experimental models have shown that a loss of GLT‐1 is involved in the pathological features of AD.^[^ ^68^, ^69^ ^]^ Accordingly, restoring GLT‐1 protein expression and function may reduce excitotoxicity and provide therapeutic benefits in AD. Ceftriaxone was the first drug to be shown to induce GLT‐1 expression via transcriptional activation.^[^ ^51^ ^]^ Ceftriaxone was previously reported to be administered mainly intravenously and intranasally. The Drugbank database shows that ceftriaxone has a blood‐brain barrier penetration of 97.48%, indicating that the drug can enter the CNS. Regarding its pharmacokinetics, ≈85%–95% of ceftriaxone is bound to plasma proteins and has an elimination half‐life in plasma of ≈6–9 h.^[^ ^70^ ^]^ However, the clinical use of ceftriaxone is limited by its high risk of gallstones (>40% in children) and its broad‐spectrum antimicrobial properties that disrupt the gut‐brain axis.^[^ ^71^, ^72^ ^]^ Although the drug increases the protein levels of GLT‐1 in vitro, ceftriaxone had limited therapeutic efficacy in chronic epilepsy^[^ ^73^ ^]^ and AD.^[^ ^74^ ^]^ Pharmacological restoration of GLT‐1 expression using LDN‐212320 was reported to attenuate cognitive deficits in a mouse model of AD.^[^ ^68^ ^]^ LDN‐212320 enhances the translation of GLT‐1, but does not affect the half‐life of the protein.^[^ ^75^ ^]^ Molecular weight of LDN‐212320 is 293.4 Da, which theoretically has a certain passive diffusion potential. GLT‐1 in the hippocampus and anterior cingulate cortex was significantly up‐regulated after intraperitoneal injection of LDN‐212320, suggesting that the drug did reach the brain tissue, which indirectly confirms that LDN‐212320 acts in the CNS.^[^ ^76^ ^]^ The side effects of LDN‐212320 are small but limited to animal experiments, and no acute toxicity or lethality has been detected in vivo tests in mice.^[^ ^77^ ^]^ However, further research is required to determine whether increasing the expression of GLT‐1 provides beneficial effects in humans.^[^ ^75^ ^]^ One possible explanation for the potential ineffectiveness of GLT‐1 is that excessive degradation of GLT‐1 in disease conditions neutralizes the effects of increasing GLT‐1 expression. We speculate that inhibition of GLT‐1 degradation might more effectively treat excitotoxicity than promote GLT‐1 expression. Therefore, the data in this study indicate that inhibition of excessive degradation of GLT‐1 by targeting PEDF may provide an alternative, more feasible treatment strategy to restore, or rather preserve, physiological levels of GLT‐1 in neurological disorders.

Beyond improving behavioral deficits, PEDF treatment also attenuated key pathological features of AD. Our data show that AAV‐*Pedf* significantly reduced both amyloid‐β plaque deposition and phosphorylated tau levels in AD mice (Figures S8I and S9A–C, Supporting Information). These results suggest that PEDF confers multifaceted benefits—rescuing cognitive function while simultaneously targeting molecular hallmarks of AD. This dual action highlights its translational potential as a promising therapeutic strategy beyond conventional single‐target approaches. Collectively, our findings indicate delivery of the *SERPINF1* gene or protein therapy could represent a promising strategy to prevent excitotoxicity, especially in the early stage of disease. Targeting these novel functions of PEDF may provide a strategy to restore physiological glutamate homeostasis as an intervention against neurodegenerative diseases associated with excitotoxicity.

## 4 Experimental Section

### Ethics Statements

Human materials used in this study were approved by the Institutional Research Ethics Committee at Zhongshan People's Hospital, Zhongshan Third People's Hospital, Guangdong Provincial People's Hospital, and Sun Yat‐sen Memorial Hospital. All participants provided written informed consent. This study was approved by the Medical Ethics Committees of Zhongshan School of Medicine, Sun Yat‐sen University (Approval number: ZZSOM‐MEC‐2021‐072). All animal experimental protocols were approved by the Ethics Committee of Experimental Animals of Sun Yat‐sen University in accordance with Institutional Animal Care and Use Committee guidelines for animal research (Approval number: SYSU‐IACUC‐2022‐B1862). The authors are responsible for the accuracy of the statements provided in the manuscript.

### Human Subjects

A total of 75 control samples without dementia were obtained from Zhongshan People's Hospital (*n* = 50), Zhongshan Third People's Hospital (*n* = 14), and Sun Yat‐sen Memorial Hospital (*n* = 11). 85 dementia subjects, including 19 AD patients, were obtained from Zhongshan Third People's Hospital (*n* = 41, including 6 AD samples), Guangdong Provincial People's Hospital (*n* = 26, including 12 AD samples), and Sun Yat‐sen Memorial Hospital (*n* = 19, including 1 AD samples). All patients with AD met the National Institute of Neurological and Communicative Disorders and Stroke‐Alzheimer's Disease and Related Disorders Associations criteria for probable AD and the National Institute on Ageing‐Reagan Institute criteria for high likelihood of AD. The collection was in strict agreement with the institutionally approved guidelines, and written informed consent was required from all participants. The study was performed in accordance with the Declaration of Helsinki (ethical principles for medical research involving human subjects). The clinical characteristics of the subjects are presented in Table S1 (Supporting Information). Scores for the MMSE and MoCA were extracted from clinical notes. These examinations were performed owing to concern for cognitive problems as part of clinical care and often recorded within neuropsychiatric evaluations.

### Animals

All animal experimental protocols were approved by the Ethics Committee of Experimental Animals of Sun Yat‐sen University in accordance with the Institutional Animal Care and Use Committee guidelines for animal research. Four to five mice were socially housed and maintained under standard housing conditions on corn cob litter in a temperature‐(23 ± 1 °C) and humidity‐(40%) controlled animal room. All mice were maintained under a 12 h light/dark cycle (lights on at 07:30, off at 19:30) with food and water ad libitum. The sample size was determined based on literature from the same field, and in consideration of the animal size limit due to animal welfare requirements and animal experimental ethical code. Although no systematic randomization method was used, animals were assigned randomly to each group. Wild‐type C57BL/6J mice were obtained from the Institute of Experimental Animals of Sun Yat‐sen University (Guangzhou, China). APP/PS1 and 5 × FAD mice were purchased from Jackson Laboratories (Stock No. 34829‐JAX and No. 006554, USA). The construction of *serpinf1* knockout model mice (*serpinf1*‐KO, *serpinf1* ^−/−^) (C57BL/6J background) referenced reported studies.^[^ ^78^ ^]^ *serpinf1* ^−/−^ mice were generously provided by Professor Jian‐Xing Ma (Department of Biochemistry, Wake Forest University School of Medicine, USA). *serpinf1* ^−/−^ 5 × FAD mice were generated by crossing 5 × FAD mice with *serpinf1* ^−/−^ mice. Only male mice with a normal appearance and weight were used for all tests. All behavioral tests were conducted by experimenters blinded to the experimental groups. Exclusion of experimental mice is strictly based on the following criteria (Internationally accepted principles of exclusion of laboratory animals):^[^ ^79^ ^]^ a) Surgical complications (injection site infection or cranial injury); b) Abnormal behavioral manifestations (>50% reduction in voluntary activity or seizures); c) Failure of tissue sampling (incomplete cerebral perfusion or sample damage); d) Outliers (Grubbs test, *α* = 0.05). Intraoperatively, the mice were kept warm and protected from burns using a small electric blanket and a small LED surgical baking lamp, which were maintained until the mice awoke. After surgery, the wounds were sutured and sterilized with iodine, and the mice were placed in a standard SPF feeding environment with high‐protein soft food and 5% dextrose water for nutritional supplementation. The mice were housed individually to observe the wound healing and the status of the mice, and if infected, they required further debridement or antidote administration, and in addition, the degree of pain of the mice was assessed by behavioral assessment, and analgesic drugs, such as non‐steroidal anti‐inflammatory drugs (NSAIDs) were administered as necessary, and if the mice reached the endpoint index, euthanasia was performed.

### Virus Injection

Before surgery, mice were anesthetized with 1.25% avertin (250 mg kg^−1^) and fixed in a stereotactic frame (RWD, China). The virus was stereotaxically injected into the unilateral or bilateral hippocampus (AP: −2.18; ML: ± 2.03; DV: −1.65 mm, where AP denotes anteroposterior from bregma, ML denotes mediolateral from the midline, and DV denotes dorsoventral from the brain surface). The adeno‐associated virus (AAV) serotype 9 for PEDF overexpression was designed and constructed by OBiO Technology (Shanghai, China). Briefly, mice were stereotactically injected with AAV expressing pAAV‐CMV‐*SERPINF1*‐3×Flag‐P2A‐mNeonGreen‐tWPA (designated as AAV‐*serpinf1*) or pAAV‐CMV‐3×Flag‐P2A‐mNeonGreen‐tWPA (designated as AAV‐con) at a dose of 2.0 ×  10^12^ viral genomes (vg), with 0.6 µL injected into each hippocampal hemisphere. This injection was administered once, and the phenotypic analysis was conducted four weeks later. rAAV‐h*syn*‐iGluSnFR was obtained from Brain Case (Shenzhen, China), and the virus was injected into the left hippocampus of mice in a volume of 0.4 µL with a titer of 1 × 10^13^ vg mL^−1^. The viruses were all injected using a 2.0 µL microsyringe (Hamilton, USA) at a rate of 0.02 µL min^−1^. The syringe was not removed until 10 min after the end of the infusion to allow the diffusion of the virus. The surgical incisions were sutured with absorbable sutures and smeared with antiseptic. Lastly, the mice were placed on an electric blanket to prevent a drop in body temperature after removal from the fixation apparatus, and then returned to the cage after the mice recovered from waking.

### Cell Lines and Cell Culture

All cells were grown and maintained at 37 °C, 5% CO_2_. Primary astrocytes and neurons were isolated and cultured as previously described, with slight modifications. In brief, mouse pups at P_0_ were sacrificed by decapitation, and the brain was collected in Hank's Balanced Salt Solution (HBSS). Olfactory bulb and cerebellum were removed, and the meninges were peeled off. The tissue was grossly minced, and incubated with 0.25% trypsin (Thermo Fisher Scientific, USA) in 0.5 mm EDTA at 37 °C for 15 min. DMEM high glucose (Gibco, USA), supplemented with 10% FBS and 1% pen‐strep (PS) was added to the preparation at a 3:1 ratio (for neurons, Start Medium: Neurobasal Medium‐A + 1% B27 + Glutamine (Sigma, USA) 0.5 mm + Glutamate 25 µm; Culture Medium: Neurobasal Medium‐A + 1% B27 + Glutamine 0.5 mm; for astrocytes, DMEM + 10% FBS). Cells were pelleted at 1000 rpm for 5 min at room temperature (RT). The pellet was resuspended in fresh culture medium, and the supernatant cell suspension was separated by stopping at 1500 rpm in rapid centrifugation mode. Lastly, the cell suspension was centrifuged at 1000 rpm for 5 min followed by resuspension of the pellet in fresh medium and plated in a T75 cell culture flask at a density of 4.0‐5.0 × 10^6^ cells. Medium was replaced on the next day. Cells were differentiated for 7 d and medium was obtained from the American Type Culture Collection (ATCC), Manassas, VA, USA, and includes the specific catalog number ATCC CCL‐107_,_ in DMEM with 10% FBS.

### Western Blotting Analysis

Cells and mouse brain tissues were lysed in ice‐cold 1 × sodium dodecyl sulfate (SDS), supplemented with protease inhibitors (PMSF, Thermo Fisher Scientific, USA) and phosphatase inhibitor cocktails (Sigma, USA). Samples were spin at 12000 × g for 20 min at 4 °C. The supernatant was collected, and protein concentration was determined via BCA Protein Assay (KGP902, KEYGEN BIOTECH, China) following manufacturer's instructions. For Western blotting, 20–30 µg protein samples were separated on 4%–15% sodium dodecylsulfate‐polyacrylamide gel electrophoresis (SDS‒PAGE) gels and subsequently transferred onto polyvinylidene difluoride (PVDF) membranes (Millipore, Germany) in ice‐cold buffer (25 mm Tris HCl, 192 mM glycine, and 20% methanol) using a semi‐dry transfer protocol for 2 h. The membrane was incubated with primary antibodies (see Table S3, Supporting Information), overnight after blocking with 5% nonfat milk for 1 h. Membranes were then washed 3 times with TBST for 5 min at RT and incubated with secondary antibodies. After 3 washes with TBST (Tris‐buffered saline with Tween 20), the bands of membranes were visualized and quantified by the imaging system (Bio‐rad, USA). The protein expression level for each sample was normalized to β‐actin.

### Coimmunoprecipitation

Coimmunoprecipitation analyses were performed as previously described.^[^ ^80^ ^]^ Collected treated cells were lysed using RIPA Lysis Buffer (P0013D, Beyotime, China). Lysates were mixed and incubated with antibodies or IgG and Protein A/G–Sepharose beads (Beyotime, China) overnight at 4 °C. Beads were then washed with lysis buffer for 3–5 times, and bound proteins were separated with subsequent immunoblotting analysis using sodium dodecyl sulfate polyacrylamide gel electrophoresis (SDS‐PAGE).

### Immunofluorescence Staining

Brain Slices: Mice were anesthetized using 1.25% avertin and were transcardially perfused with 1× PBS (137 mm NaCl, 2.7 mm KCl, 10  mm Na_2_HPO_4_, 1.8 mm KH_2_PO_4_, pH 7.4) for 2 min, following 4% paraformaldehyde for 5 min at a rate of 10 mL min^−1^. Fixed brains were sliced using a Vibratome VT1500S (Leica, Germany) at 10 µm thickness. Slices were permeabilized with 0.1% Triton X‐100 in 1× PBS for 20 min at RT, followed by incubation with blocking buffer (2% BSA in permeabilization buffer) for 1 h at RT and then with primary antibodies in blocking buffer overnight at 4 °C. Brain slices were then washed in 1× PBS and further incubated with fluorescently labeled secondary antibodies (1:200 in blocking buffer) for 1 h at RT. After additional washes in 1× PBS, nuclei were stained for 10 min at RT with DAPI (1 µg mL^−1^ in 1× PBS). Anti‐fade solution (P36970, Invitrogen, USA) was applied for mounting. Finally, slices were mounted on microscope slides. Acquisitions were taken at a Zeiss slide scanner (AxioScan.Z1, Germany), with a 20× or 40× objective.

### Cells

Cultured cells were seeded on coverslips at the appropriate density. The cells were fixed in 4% paraformaldehyde for 30 min and permeabilized with 0.1% Triton X‐100 in 1× PBS for 10 min at RT, followed by incubation with a blocking buffer (2% BSA in permeabilization buffer) for 1 h at RT, and then with primary antibodies overnight at 4 °C. On the following day, the samples were washed and incubated with a secondary antibody for 1 h at RT. The nuclei were counterstained with DAPI. The coverslips were mounted onto glass slides with anti‐fade solution and visualized under a Leica (DM6B, Germany) fluorescence microscope.

### Dendritic Spines Density Quantification (Golgi‐Cox Staining)

To determine the density of dendritic spines, brain tissue was processed for Golgi‐cox staining followed by confocal imaging of the CA1 region dendrites and spines. Specifically, mice were terminally anesthetized with 1.25% avertin and perfused with ice‐cold 1× PBS (10 ml total volume, over 3 min). Impregnation solution preparation was prepared as follows: Solution A including 5% solution of potassium dichromate (K_2_Cr_2_O_7_, Guangzhou chemical reagent factory); Solution B including 5% solution of mercuric chloride (HgCl_2_, Guangzhou chemical reagent factory, China); Solution C including 5% solution of potassium chromate (K_2_CrO_4_, Guangzhou chemical reagent factory, China). First, mixed 5 mL solution A, 5 mL solution B, 4 mL solution C, and 10 mL distilled water to get Golgi‐Cox solution. After sufficiently mixing the solution, it was kept in the dark at RT for 3 days. After reddish precipitates form, aspirate the supernatant to get the working solution. Finally, slices were treated with a working solution, and protected from light exposure whenever possible. After 10 days of impregnation, transferred slides into the 30% sucrose (Guangzhou chemical reagent factory, China) solution for 6 h. The slides were incubated in 14% ammonia solution (Aladdin, China) for 10 min in the dark by gently shaking them. Next, the slides were dehydrated through 70%, 80%, 95%, and 100% (twice) ethanol for 5 min each, transferred to the xylol (Macklin, China) for 10 min, then mounted the sections with a permanent mounting medium. Images were obtained with a Zeiss slide scanner (AxioScan.Z1, Germany), with a 40× objective. For analysis, reconstruction of individual pyramidal neuron, dendrites lengths, Sholl intersections, and spines counting was performed using Fiji (v1.54F).

### Nissl Staining

Nissl staining was performed as previously described.^[^ ^81^ ^]^ After the hippocampus tissues fixed in 4% PFA were dehydrated in a series of graded alcohols (70–100%), the tissues were embedded in paraffin and sliced to a thickness of 10 µm using a rotary microtome. The sections were placed on a microscope slide, dried at 56 °C for 2 h, and stored at RT. For Nissl staining, sections were deparaffinized and rehydrated using xylene and graded alcohols (100%–70%), and then rinsed in distilled water. Tissue sections were incubated with Nissl Staining Solution (Beyotime, China) at RT for 10 min, and washed with distilled water and 70% ethanol twice. Sections were quickly dehydrated in 95% and 100% ethanol, cleared in xylene, and mounted with a resinous mounting medium. Images were obtained with a Zeiss slide scanner (AxioScan.Z1, German), with a 40× objective and analyzed using Fiji (v1.54F).

### RNA Extraction and Quantitative Real‐Time Polymerase Chain Reaction (qPCR) Analysis

Total RNA was extracted with Trizol reagent (Invitrogen, USA), and RNA concentration was measured by NanoDrop spectrometer. Prime Script RT reagent Kit Perfect Real Time kit (TAKARA, Japan) was used to reverse cDNA transcription. Real‐time quantitative PCR was performed using SYBR‐Green fluorescent dye (RK21203, Abclonal, China) with a Biorad CFX 96 (USA). The related primer sequences were in Table S4 (Supporting Information). Each reaction was performed in triplicate. Results were analyzed by the comparative CT method. Average CT values for each sample were normalized to the average CT values of the housekeeping gene. The differences in gene expression were calculated by the 2^−ΔΔCT^ method and were presented as the fold change.

### Small Interfering RNAs (si‐RNAs) Construction and Cell Transfection

Mouse si‐*serpinf1*, rat si‐*pnpla2*, and si‐NC control were constructed by Ribobio (Guangzhou, China). Sense Sequences: 5′‐TCACCCGACTTCAGCAAGA‐3′(si‐m‐*serpinf1*); 5′‐AGACCAUCCGUGGUUGUCUACUGAA‐3′(si‐rat‐*pnpla2*). Cells were grown to 70% confluence and transfected using Lipofectamine 3000 (Thermo Fisher Scientific, USA) according to the manufacturer's protocol. The cells were transfected for 24 h at 37 °C.

### Recombinant PEDF Treatment

For treatment with mouse PEDF (8295‐SF, R&D Systems, USA), medium containing graded concentrations of PEDF were added to appropriate densities of astrocytes or C6. Recombinant PEDF was maintained in the medium for an additional 12 h throughout the experiment.

### Measurement of Serum PEDF Level

To quantify PEDF level, the serum was centrifuged at 8000 rpm for 10 min. Serum PEDF concentrations were measured with an ELISA kit (DY1177‐05, R&D Systems, USA), following the manufacturer's instructions.

### Evaluation of Glutamate Uptake Ability

Primary astrocytes and C6 cells were treated with 100 µm L‐glutamate (Sigma, USA) and incubated for 4 h at 37 °C. At each time point, the concentration of glutamate in the medium was measured with a glutamate assay kit (BC1585, Solarbio life sciences, China) according to the manufacturer's protocol, and the glutamate uptake ability was calculated as follows: concentration at each time point‐concentration 0 min.

### Behavioral Tests

Open Field: The open‐field arena was a light blue box (50 × 50 cm area, 40 cm high walls) illuminated with an ambient light (15 lux), placed inside a sound‐insulated chamber. The mice were placed into the arena facing a wall and allowed to explore the arena for 10 min. The total distance traveled, as well as the distances traveled and time spent in the 20 × 20 cm center zone were tracked and analyzed by the TopScan (Clever Sys. Inc, USA) software.

### Novel Object Recognition

The mice were habituated to the experimental room for 3 consecutive days before the training phase, during which the mice were allowed to freely explore the light blue testing arena (50 × 50 cm area, 40 cm high walls) with dim light (15 lux) for 10 min per day. For the training, two identical objects were placed in 2 corners of the arena 10 cm from the wall. After 5 min of freely exploring the objects, the mice were placed in individual cages and were not returned to their original cages, as this might affect the behavior of the remaining mice to be tested. Memory of the object was tested 60 min after training. One of the familiar objects was replaced by a novel object that was different from the familiar ones, and the locations of these objects were unchanged. The percentage of time that the mice spent exploring the novel object in 3 min was calculated as the capacity for memory of the object. All objects were previously screened, and the mice showed no significant preference for these objects. The arena and objects were wiped clean with a paper towel soaked in 75% ethanol and dried thoroughly after each test session. The discrimination index is calculated as time in the novel object /total time in the novel object and familiar object × 100% and given as a percentage.

### Y‐Maze

Y‐maze testing was adapted from published protocols.^[^ ^82^ ^]^ The Y‐maze apparatus consisted of 3 arms joined in the middle to form a Y shape. The walls of the arms were 10 cm high, and each was marked with a single large black letter to serve as a spatial landmark and clue. With one arm of the maze closed, mice were allowed to explore the other two arms for 15 min before being returned to their home cage. 60 min later, mice were returned to the Y‐maze and allowed to explore all three arms for 5 min while being video recorded. The time spent in each arm was quantified. The discrimination index is calculated as time in the novel arm/total time in the novel arm and familiar arm × 100% and given as a percentage.

### Morris Water Maze

The Morris water maze test was performed with minor adjustments as previously described.^[^ ^82^ ^]^ Spatial memory testing was conducted in a circular tank (diameter 1.2 m) filled with opacified water at 22 °C. The water tank was dimly lit and surrounded by a black curtain. The maze was virtually divided into four quadrants, with one containing a hidden platform (diameter 10 cm) that was submerged 1 cm below the water level. Four prominent cues were placed outside the maze as spatial references. Before testing phase, mice were allowed to acclimate to the testing room for 3 days. Mice were placed in the water facing the tank wall at different start positions across trials in a quasi‐random fashion to prevent strategy learning. Mice were allowed to search for the platform for 70 s. If the mice did not find the platform, they were guided towards it where they remained for 20 s. Each mouse underwent four trials (one from each start position) daily for 5–7 consecutive days. Each trial required an interval of at least 2 min. After each trial, the mouse was dried and placed back into its cage until the start of the subsequent trial. All mouse movements were recorded by the computerized tracking system TopScan (Clever Sys. Inc, USA), which calculated distances moved and time required to reach the platform (escape latency), along with swim speed. The spatial probe trial was conducted 24 h after the last training session (on day 6 or day 8). For the probe trial, the platform was removed, and mice were allowed to swim for 70 s. The following events are required to be recorded: the duration of the mice spent in the quadrant where the platform was previously located, the number of crossings over the location where the platform was initially positioned, and the time taken by the mice to reach the original platform location for the first time. Data were calculated as time spent in the quadrant where the original platform was located/70 s × 100% and is given as a percentage.

### Fear Conditioning

The fear conditioning test was based on the Pavlovian fear conditioning paradigm, in which a previously neutral (conditioned) stimulus was paired with an aversive (unconditioned) stimulus, which resulted in a measurable state of fear. Fear conditioning training was conducted in a chamber (28 × 28 × 30 cm) with black walls and a speaker. The floor was made of stainless‐steel rods spaced at 0.5 cm and connected to a shock delivery apparatus. The conditioning apparatus was placed in a sound‐reducing chamber (typical background noise was 10 db) illuminated with an ambient light (100 lux). Mice were placed in the apparatus freely for 2 min, during which the light intensity and background noise were kept constant. Following a 2 min acclimation period (baseline activity), mice were exposed to a 30 s tone (40 db) that co‐terminated with a 2 s foot‐shock (0.50 mA). The duration between pairings was 90 s and lasted 3 times. After the last pairing, the mice were left in the conditioning chamber for 90 s. Be cautious not to return the mice to their initial cages since the mice were irritable after training, which may affect the behavior of the remaining mice to be tested. Contextual memory: The day following the conditioning session mice were returned to context under identical conditions as their training session (100 lux, 10 db) without any stimuli, including tones and foot‐shock. Cued memory: The following day after the contextual memory test, mice were placed in an altered context (including changes in visual and odour cues) for 3 min of free movement. The light was kept at 100 lux while the background noise was changed to 5 db, and then the mice were given a 40 db tone for 3 min without foot‐shock. Freezing was measured concerning the threshold of baseline activity. Movement was tracked using FreezeFrame software (Actimetrics, COULBOURN, USA) and freezing calculated as a percentage.

### *Ex Vivo* Glutamate‐Imaging of Acute Hippocampal Slices

The mice were anesthetized with 1.25% avertin. The brain was quickly removed from the skull after decapitation, and 400 µm coronal slices containing the hippocampus were prepared using a Leica LS1500s vibrating microtome in ice‐cold oxygenated artificial cerebrospinal fluid (ACSF) containing (in mm) (Sigma, USA): 2.5 KCl, 220 sucrose, 1 NaH_2_PO_4_, 1.3 CaCl_2_, 2.5 MgSO_4_, 26 NaHCO_3_, and 10 glucose. The slices were transferred to a chamber and held at 35 °C for 30 min and at RT for 1 h. The slices were then kept at RT until recording. A slice was transferred to the recording chamber that was constantly perfused with oxygenated ACSF at 35 °C. The slice was viewed under an Olympus FVMPE‐RS two‐photon microscope (Olympus, Japan) equipped with a 25× water immersion objective and a laser wavelength of 920 nm. Glutamate imaging was recorded in the stratum radiatum of hippocampal CA1. The baseline activities were recorded during a 10 min period. After a hypoxic stimulus, i.e., the oxygenated ACSF was replaced with 95% N_2_‐5% CO_2_ ACSF, the relative fluorescent value F was recorded and quantified. Time‐series images were captured with a scanning thickness of 150 µm and a 10 µm interval between scans. The total scanning time was at least 40 min, with each time‐lapse sequence lasting ≈30 s or 1 min. The scanning resolution of images was set at 1024 × 1024 pixels. The fluorescence signals were normalized within each mouse by calculating the delta fluorescence/fluorescence (ΔF/F_0_) as (F–F_0_)/F_0_, where F_0_ is the baseline fluorescence signal averaged before the onset of the stimulus event. The positions of the recording sites in mice used in the in vitro recording experiments were verified after recording.

### *Ex Vivo* Electrophysiology

Whole‐cell recordings were performed as described previously.^[^ ^83^, ^84^ ^]^ Briefly, mice were anesthetized with 1.25% avertin and transcardially perfused with ice‐cold solution containing the following (in mm): 212.7 sucrose, 3 KCl, 1.25 NaH_2_PO_4_, 3 MgCl_2_, 1 CaCl_2_, 26 NaHCO_3_, and 10 dextrose, bubbled with 95% O_2_ / 5% CO_2_. Coronal slices from hippocampus (≈400 µm) were prepared using a tissue slicer (Vibratome 3000, Leica, German) in ice‐cold dissection buffer, which is the same as perfusion solution. The slices were immediately transferred to ACSF at 35 °C for 30 min before recordings. The recipe of ACSF was similar to the dissection buffer, except that sucrose was replaced with 124 mm NaCl, and the concentrations of MgCl_2_ and CaCl_2_ were changed to 1 and 2 mm, respectively. All recordings were performed at 28°C–30 °C. Pyramidal cells in CA1 were identified visually under infrared differential interference contrast optics based on their pyramidal somata and prominent apical dendrites. Recording electrodes with a resistance of 4–8 MΩ were pulled from borosilicate glass capillaries (1.5 mm outside diameter [OD]) using a P‐97 electrode puller. The internal pipette solution contained (in mm) (Sigma, USA) 125 cesium methanesulfonate, 5 CsCl, 10 HEPES, 0.2 EGTA, 1 MgCl_2_, 4 Mg‐ATP, 0.3 Na‐GTP, 10 phosphocreatine, and 5 QX314 (pH 7.40, 290 mOsm). The recordings were obtained using an Integrated Patch‐Clamp Amplifier (Sutter Instrument, Novato, CA, USA) controlled by Igor 7 software (WaveMetrics, Portland, OR, USA) filtered at 5 kHz and sampled at 20 kHz. Igor 7 software was also used for acquisition and analysis. mEPSCs were recorded in the presence of 1 µm tetrodotoxin (Aladdin, China) and 20 µm bicuculine (Sigma, USA).

### Extracellular Field Recordings

Hippocampal brain slice preparation was performed in this study using 9‐month‐old wild‐type (WT) and *serpinf1* knockout (KO) mice. Mice were anesthetized with 1.25% avertin and transcardially perfused with cold cutting solution. After perfusion, brains were rapidly removed and immersed in a cold high‐sucrose cutting solution containing (in mm): 200 sucrose, 12 MgSO_4_, 10 glucose, 0.2 CaCl_2_, 2 KCl, 1.3 NaH_2_PO_4_, 26 NaHCO_3_, and 1.8 ascorbic acid. Coronal hippocampal slices (350 µm) were obtained using a vibrating microtome (Leica VT‐1200S) and incubated in ACSF at 32 °C for 30 min, followed by an additional 1 h at RT to facilitate recovery. The ACSF solution was composed of (in mm): 126 NaCl, 3 KCl, 1.25 NaH_2_PO_4_, 26 NaHCO_3_, 10 glucose, 2 CaCl_2_, 1 MgSO_4_, and 1.8 ascorbic acid. Both cutting solution and ACSF were continuously bubbled with a 95% O_2_/5% CO_2_ gas mixture to maintain physiological pH and tissue viability throughout the slicing and recording procedures.

LTP recordings were performed in the stratum radiatum of the CA1 region.^[^ ^85^ ^]^ A bipolar stimulating electrode was placed in the Schaffer collateral pathway, and fEPSPs were recorded using glass micropipettes (1–4 MΩ) filled with ACSF. Baseline synaptic responses were evoked every 30 s, and the average of two consecutive fEPSP responses was taken at each time point for analysis. Input‐output curves were generated by incrementing the stimulus intensity. A stimulation strength evoking 1/3‐1/2 of the maximal fEPSP amplitude was used for baseline and subsequent LTP induction. After achieving a stable baseline for at least 20 min, high‐frequency stimulation (HFS) was applied, consisting of two trains of 100 Hz stimulation separated by a 10 s interval. Recordings were continuous for 60 min following HFS. LTP magnitude was quantified by normalizing each fEPSP amplitude to the average baseline value. The final LTP level was calculated by averaging the fEPSP amplitude recorded during the last 10 min of the 60 min post‐HFS period, which was used to plot a box plot of percentage change.

### Seizure Susceptibility Assessment Upon Kainic Acid Administration

Mice were injected intraperitoneally with 2 mg kg^−1^ kainic acid (KA, HY‐N2309, MCE, USA) in saline, and their behavior was videorecorded and blindly scored for 2 h according to the modified Racine scale. Data were analyzed by plotting the highest behavioral score assigned to each animal over 30 min intervals, for the 2 h of observation.

### Library Preparation and RNA Sequencing

The library preparation and RNA sequencing were carried out by SEQUMED (Guangzhou, China). The Ultrapure RNA Kit (CWBIO, China) was used to amplify the RNAs and prepare the cDNA library. Then the libraries were loaded onto an Illumina HiSeq instrument (Illumina, San Diego, CA, USA) for paired‐end 150 bp sequencing. The raw sequencing reads were filtered for removing adapter sequencing, primers, polyA tail sequences, and reads of low‐quality bases. The quality of the reads was evaluated using Trimmomatic. Then the processed sequences were aligned to the reference genomes based on BWT and FM index using HISAT2. DESeq2 was used to normalize the raw counts and perform differential expression analysis. Differentially expressed genes (DEGs) were defined as *p* < 0.05 and an absolute value of FoldChange > 2. The heatmap and volcano plot of the DEGs were generated using the pheatmap package in R. Functional enrichments were performed with the gene‐set enrichment analysis (GSEA) to identify KEGG pathways and gene ontology that were differentially enriched in the KO group and the control counterparts. Gene expression dataset of different brain regions from AD patients (GEO accession number: GSE48350) was used for comparison with the DEGs identified in the current study.

### Postmortem Brain Tissue Bioinformatics Analysis

The GEO dataset (GSE36980) was downloaded from the Gene Expression Omnibus (GEO) database, consisted of hippocampus from 7 AD patients and 10 non‐AD controls (Sample GSM4764672 was excluded). These brain samples were donated for the Hisayama study. The dataset of hippocampus in the MergedGEO included GSE28146, GSE29378, GSE36980, GSE48350, and GSE5281. All differential expression results were adjusted for the age and sex of the samples.

### Statistical Analysis

The statistical methods used for each analysis are described in the figure legends. Briefly, paired Student's *t* test and paired Wilcoxon test were used to determine significant differences for the comparison between two paired groups. The unpaired Student's *t* test was used to detect whether there were significant differences between two independent groups. One‐way ANOVA combined with Tukey's multiple comparison method was used to conduct pairwise comparisons among multiple independent groups (≥ 3). Two‐way ANOVA combined with Tukey's or Bonferroni's multiple comparison method was used to perform pairwise comparisons among multiple groups with two factors. All statistical tests were two‐sided, and the statistical results are presented as the mean ± S.E.M. All experiments were repeated at least three times, yielding consistent results. Representative outcomes and images are presented. Statistical analyses were performed with GraphPad Prism (version 8.0.1). For correlation analysis, the correlation was computed using Pearson correlation coefficients. *p* values of 0.05 or less were considered to denote significance. Statistical significance was set at ^*^ *p* < 0.05, ^**^ *p* < 0.01, ^***^ *p* < 0.001, and ^****^ *p* < 0.0001. n.s., no significance.

## Conflict of Interest

The authors declare no conflict of interest.

## Author Contributions

W.‐W.Q., G.‐Q.G., X.Y. and B.‐X.L. conceived and directed the project; J.‐H.S., and W.‐W.Q. designed the experiments and wrote the manuscript. J.‐H.S., Q.‐L.T., Z.Z., W.‐T.X., S.‐F.Z., Z.‐M.L. and H.‐M.L carried out the experiments; J.‐H.S., J.‐H.W., Q.‐L.T. and Y.‐L.L. analyzed and interpreted the data; T.‐X.G., Z.‐Z.F. and T.Z. revised the manuscript. All authors contributed to the article and approved the submitted version.

## Supporting information



Supporting Information

## Data Availability

The data that support the findings of this study are available from the corresponding author upon reasonable request.
