# Supplementary material for: Pigment Epithelium‐Derived Factor Deficiency Impairs Hippocampal Glutamate Homeostasis and Cognitive Function by Downregulating Astrocytic GLT‐1
Source: Adv Sci (Weinh). 2025 Sep 14;12(45):e00402. doi: 10.1002/advs.202500402 (PMC12677611; doi:10.1002/advs.202500402)
Supplement: Supplementary file 1 — Supporting Information [file ADVS-12-e00402-s001.pdf]

Supporting Information

Pigment Epithelium-Derived Factor Deficiency Impairs Hippocampal Glutamate Homeostasis and Cognitive Function by Downregulating Astrocytic GLT-1

Jin-Hui Shi, Qi-Long Tang, Jin-Hong Wang, Yan-Lan Long, Sai-Feng Zhao, Zhen Zhao, Wan-Ting Xie, Zi-Ming Li, Hao-Ming Lu, Tian-Xiao Gao, Zhen-Zhen Fang, Ti Zhou, Bo-Xing Li, Xia Yang*, Guo-Quan Gao* and Wei-Wei Qi**

Supplementary materials

Pigment Epithelium-Derived Factor Deficiency Impairs Hippocampal Glutamate Homeostasis and Cognitive Function by Downregulating Astrocytic GLT-1

Jin-Hui Shi, Qi-Long Tang, Jin-Hong Wang, Yan-Lan Long, Sai-Feng Zhao, Zhen Zhao, Wan-Ting Xie, Zi-Ming Li, Hao-Ming Lu, Tian-Xiao Gao, Zhen-Zhen Fang, Ti Zhou, Bo-Xing Li, Xia Yang*, Guo-Quan Gao*, Wei-Wei Qi**

J.H. Shi, Q.L. Tang, J.H. Wang, Y.L. Long, S.F. Zhao, Z. Zhao, W.T. Xie, Z.M. Li, H.M. Lu, T.X. Gao, Z.Z. Fang, T. Zhou, B.X. Li, X. Yang, G.Q. Gao, W.W. Qi

Department of Biochemistry and Molecular Biology, Zhongshan School of Medicine, Sun Yat-sen University, Guangzhou 510080, China

E-mail: shijh9@mail2.sysu.edu.cn; qiww3@mail.sysu.edu.cn

Supplemental Figures and Legends:

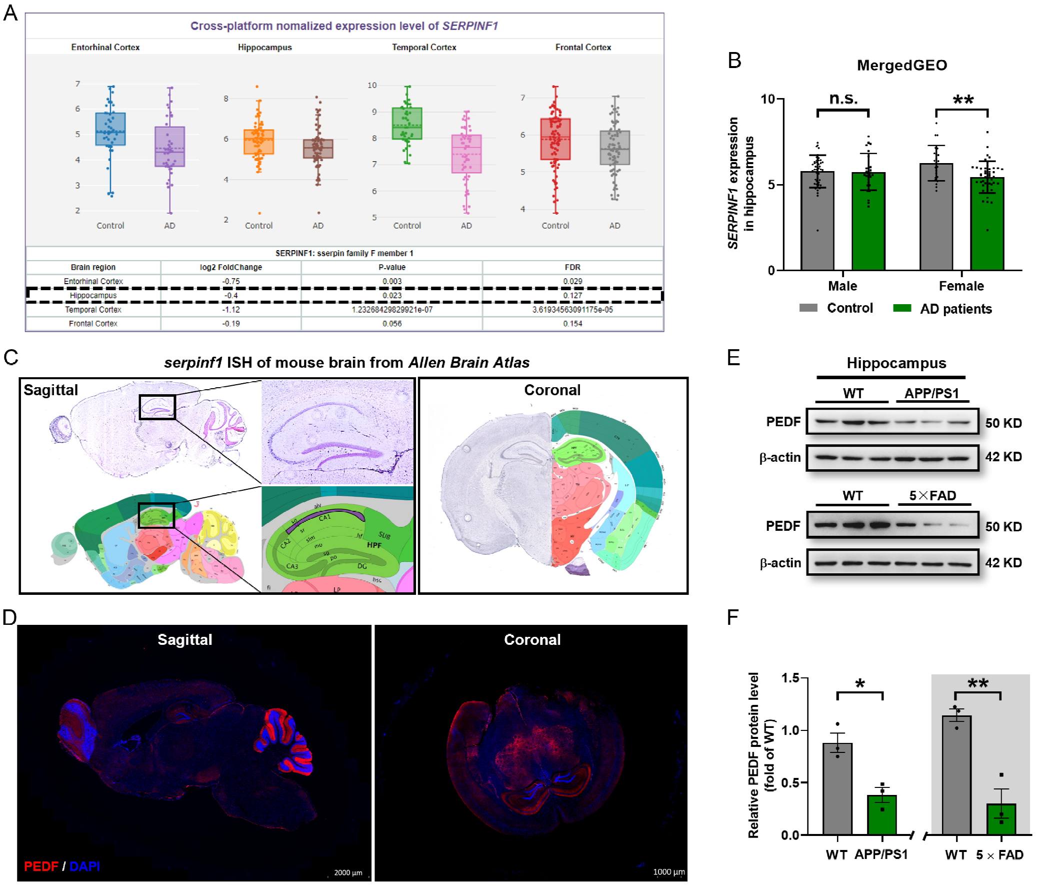

Figure S1. PEDF is decreased in hippocampus of AD patients and AD mouse model. A, *SERPINF1* mRNA level analysis results in different brain regions including hippocampus from AD patients and controls were obtained from AlzData database (https://www.alzdata.org). **B**, The normalized *SERPINF1* transcripts in the hippocampus of AD patients (29 males and 45 females) and healthy controls (43 males and 23 females), the dataset was merged from GSE28146, GSE29378, GSE36980, GSE48350, and GSE5281. **C**, The *serpinf1* ISH of mouse brain from Allen Brain Atlas (https://mouse.brain-map.org). **D**, Representative images of PEDF staining in the sagittal and coronal brain sections of C57B6/J mice. Scale bars: 2000 μm (sagittal); 1000 μm (coronal). **E** and **F**, Western blotting analysis of PEDF in

the hippocampus of AD model mice including 6-mo-old APP/PS1, and 8-week-old 5 × FAD mice (E). Protein levels were normalized to β-actin (F), $n = 3$ mice per group. Data are presented as the mean ± S.E.M. Comparison by two-tailed unpaired Student's *t*-test unless otherwise indicated. $^{*}P < 0.05$, $^{**}P < 0.01$; n.s., not significant.

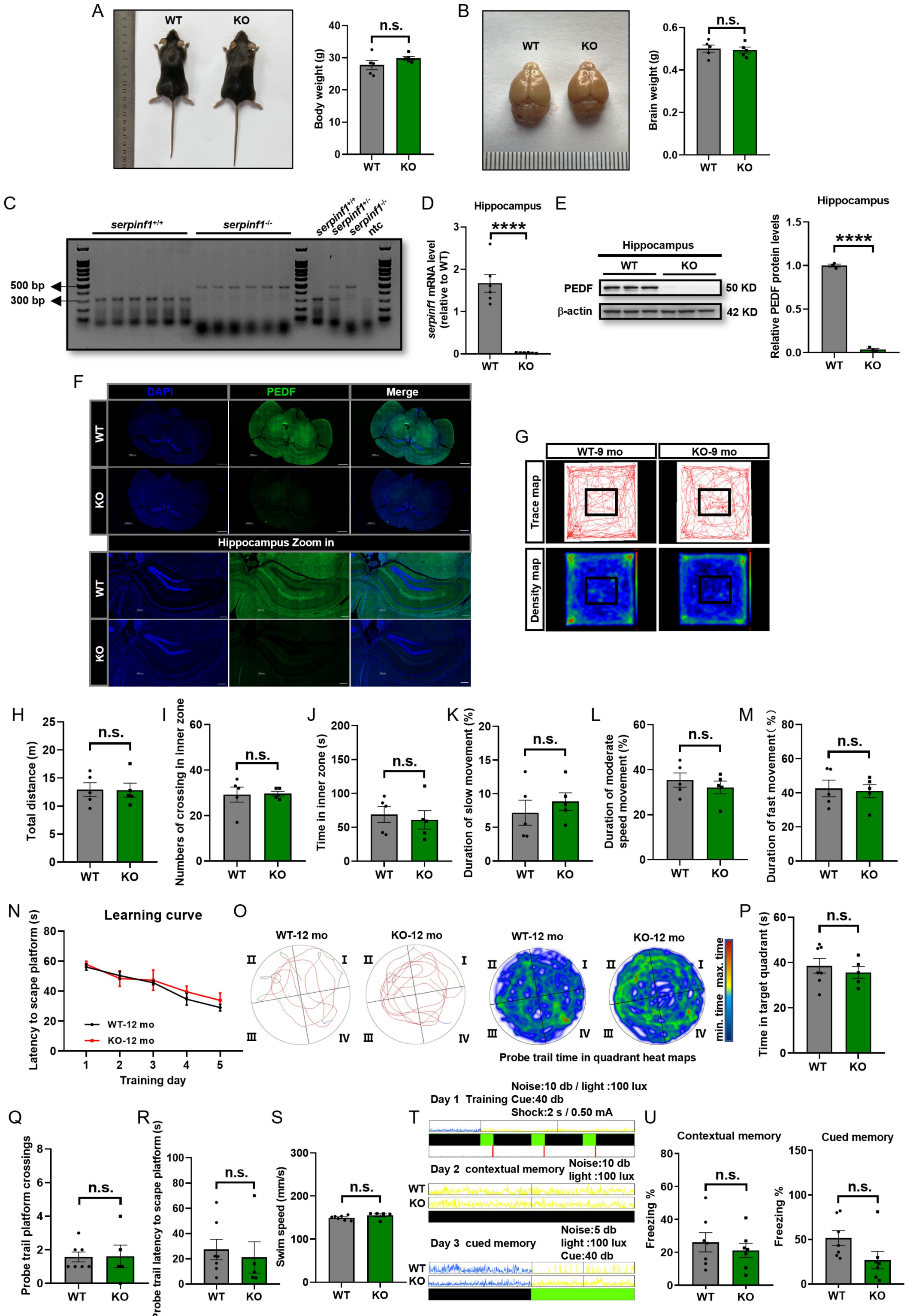

Figure S2. Identification and behavioral testing of *serpinf1*$^{-/-}$ mice and WT controls. A and **B**, Representative image and quantification of body weight (A) and brain weight (B) of KO and littermate WT controls (n = 5 mice per group). **C**,

Representative image of agarose gel electrophoresis of mouse tail DNA sample. **D**, Gene expression of *serpinf1* mRNA in the hippocampus of WT and KO mice (n = 6 mice per group). **E**, Western blotting analysis of PEDF in the hippocampus of WT and KO mice (left panel). Protein levels were normalized to β-actin (right panel, $n = 3$ mice per group). **F**, Representative images of PEDF staining in the brain sections of WT and KO mice. Scale bars: 200 μm (bottom, magnified view of the top image); 1000 μm (top). **G-M**, Representative track and heat map images (G), total moving distance (H), number of inner zone crossings (I), duration time in the inner zone (J), duration of slow (K), moderate (L) and fast movement (M) by 9-mo-old WT and KO mice in the OFT (n = 5 mice per group). **N**, Time to reach hidden platform by 12-mo-old KO and WT in the MWM plotted across training days. Two-way ANOVA with the Bonferroni's multiple comparison. **O-S**, Representative track and heat map images of mice (O), time spent in target quadrant (P), platform crossing count (Q), latency of first time to enter the target (R) and mean swimming speed (S) in the probe trial of the MWM test ($n = 7$ mice for the WT group and $n = 5$ mice for the KO group). **T**, Schematic diagram of the fear conditioning (FC) test by 12-mo-old KO and WT. **U**, Percent time of freezing in contextual memory test (left panel) and cued memory test (right panel) ($n = 7$ mice per group). Data are presented as mean ± S.E.M. Comparison by two-tailed unpaired Student's *t*-test unless otherwise indicated. ****$P < 0.0001$; n.s., not significant.

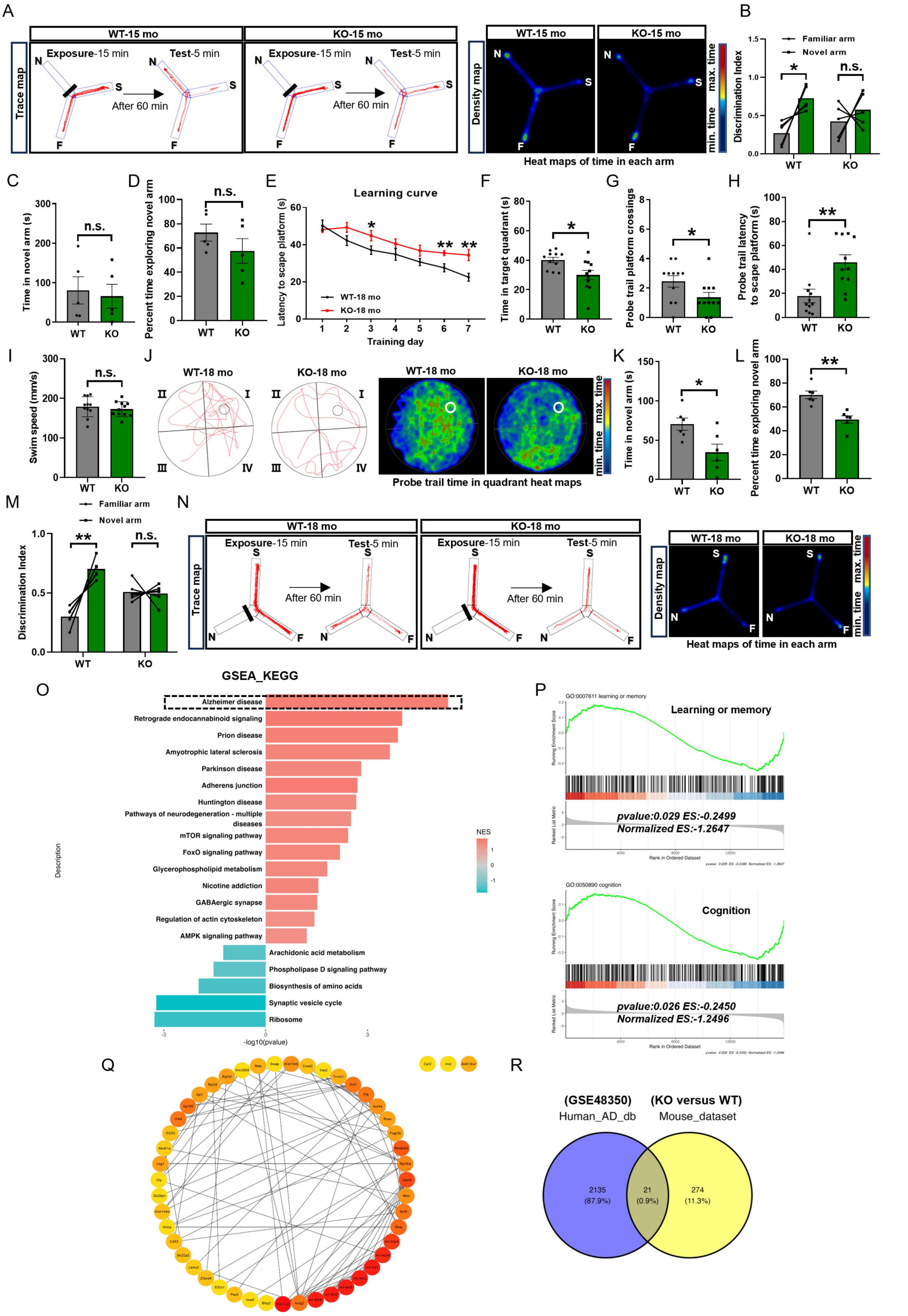

Figure S3. Long-lasting behavioral testing and the alterations of the hippocampal transcriptomic signatures of *serpinf1*$^{-/-}$ mice. A, Schematic diagram, representative track and heat map images by 15-mo-old KO and WT

in the Y-maze test. **B**, Discrimination index in Y-maze test (n = 5 mice per group). Calculation similar to the NOR test discrimination index. **C** and **D**, Time spent in novel arm (C), and percent time exploring the novel arm to total time exploring novel arm and familiar arm (D). **E**, Time to reach hidden platform by 18-mo-old KO and WT in MWM plotted across training days. **F-J**, Time spent in target quadrant (F), platform crossing count (G), latency of first time to enter the target (H), mean swimming speed (I), and representative track and heat map images of mice (J) in the probe trial of the MWM test (n = 11 mice per group). **K-N**, Time spent in novel arm (K), percent time exploring the novel arm to total time exploring novel arm and familiar arm (L), discrimination index (M), and representative track and heat map images (N) by 18-mo-old KO and WT mice in the Y-maze test (n = 6 mice per group). **O**, Top enriched (NES > 0) and de-enriched (NES < 0) gene sets in the KEGG revealed by GSEA. **P**, GSEA of the learning and memory, cognition hallmark pathway. **Q**, Co-expression network of Top50 proteins of DEGs. **R**, A Venn diagram represents the number of overlapping genes between KO mouse dataset and human AD dataset (GSE48350). Data are presented as mean ± S.E.M. Two-tailed paired Student's t-test (B), (M); Two - way ANOVA with the Bonferroni's multiple comparison (E); Comparison by two-tailed unpaired Student's t-test unless otherwise indicated. $^{*}P < 0.05$, $^{**}P < 0.01$; n.s., not significant.

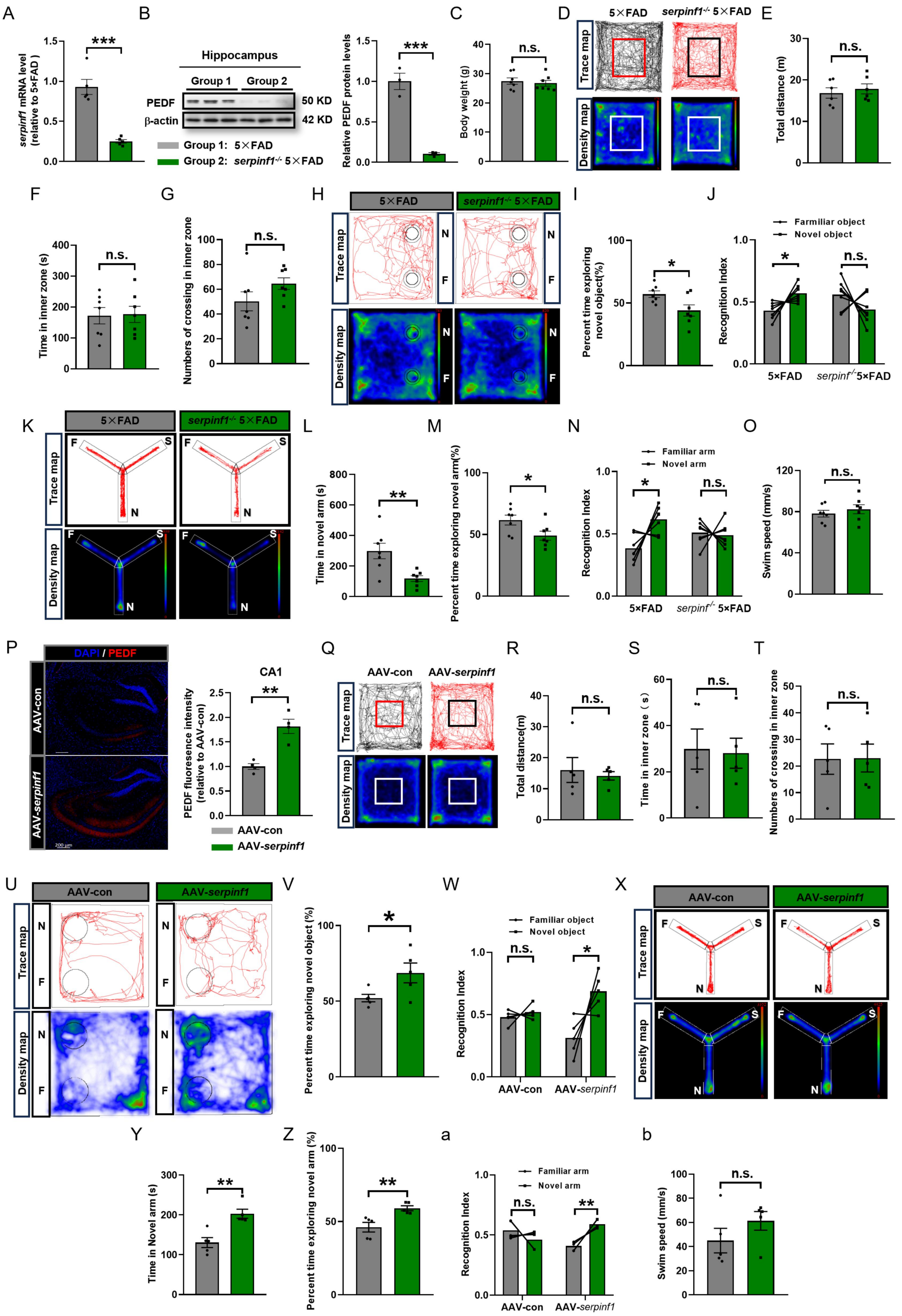

Figure S4. Identification and behavioral testing results of *serpinf1*$^{-/-}$ 5 × FAD mice and AAV-*serpinf1* injected APP/PS1 mice. A, Gene expression of *serpinf1* mRNA in the hippocampus of 5 × FAD and *serpinf1*$^{-/-}$ 5 × FAD mice (*n*

= 5 mice per group). **B**, Western blotting analysis of PEDF in the hippocampus of 5 × FAD and *serpinf1*$^{-/-}$ 5 × FAD mice (left panel). Protein levels were normalized to β-actin (right panel, *n* = 3 mice per group). **C**, Quantification of body weight of 5 × FAD and *serpinf1*$^{-/-}$ 5 × FAD mice (*n* = 7 mice per group). **D-G**, Representative track and heat map images (D), total moving distance (E), duration time in the inner zone (F), number of inner zone crossings (G) by 5 × FAD and *serpinf1*$^{-/-}$ 5 × FAD mice in OF test. **H**, Representative track and heat map images by 8-week-old 5 × FAD and *serpinf1*$^{-/-}$ 5 × FAD mice in the NOR test. **I** and **J**, Quantification of percentage time spent in the novel and familiar object (I) and discrimination index (J) during the test phase (*n* = 7 mice per group). **K-N**, Representative track and heat map images (K), time spent in novel arm (L), percent time exploring the novel arm to total time exploring novel arm and familiar arm (M), discrimination index (N) by 5 × FAD and *serpinf1*$^{-/-}$ 5 × FAD mice in the Y-maze test. **O**, Mean swimming speed in the probe trial of the MWM test. **P**, Representative images of PEDF staining in the brain sections of AAV-con and AAV-*serpinf1* injected mice (left panel); quantification of the fluorescence intensity in CA1 (right panel, *n* = 3 mice per group). Scale bars: 200 μm. **Q-T**, Representative track and heat map images (Q), total moving distance (R), duration time in the inner zone (S), number of inner zone crossings (T) by AAV-con and AAV-*serpinf1* injected mice in OFT (*n* = 4 mice per group). **U**, Representative track and heat map images by 6-mo-old APP/PS1 mice treated with AAV-con and AAV-*serpinf1* in the NOR

test. **V** and **W**, Quantification of percentage time spent in novel and familiar object (V) and discrimination index (W) during the test phase (n = 5 mice per group). **X-a**, Representative track and heat map images (X), time spent in novel arm (Y), percent time exploring the novel arm to total time exploring novel arm and familiar arm (Z), discrimination index (a) by AAV-con and AAV-*serpinf1* injected mice in the Y-maze test. **b**, Mean swimming speed in the probe trial of the MWM test. Data are presented as mean ± S.E.M. Two-tailed paired Student's *t*-test (J), (N), (W), (a); Comparison by two-tailed unpaired Student's *t*-test unless otherwise indicated. *$P < 0.05$, **$P < 0.01$, ***$P < 0.001$; n.s., not significant.

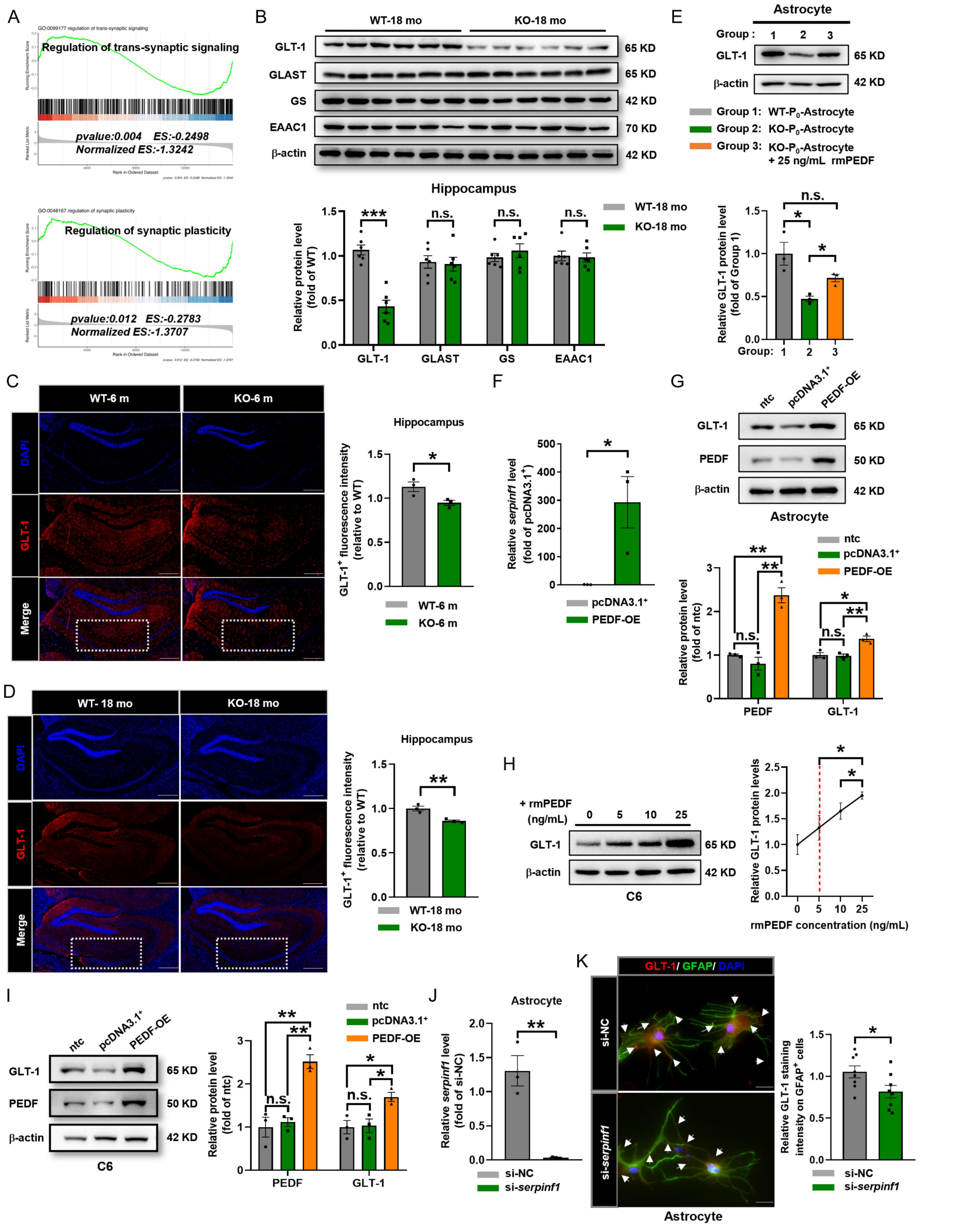

Figure S5. PEDF physiologically upregulates GLT-1 protein levels in a dosage-dependent way. A, GSEA of the regulation of trans-synaptic, regulation of synaptic plasticity hallmark pathway. **B**, Western blotting analysis and quantification of GLT-1, GLAST, GS, and EAAC1 in the hippocampus of 18-mo-old KO and WT mice ($n = 6$ mice per group). **C**, Representative images of GLT-1 in the brain sections of 6-mo-old KO and WT mice (left panel);

quantification of the fluorescence intensity of the GLT-1$^+$ area in CA1 (right panel, $n = 3$ mice per group). Scale bars: 200 μm. **D**, Representative images and quantification of GLT-1 in the brain sections of 18-mo-old KO and WT mice ($n = 3$ mice per group). Scale bars: 200 μm. **E**, Western blotting analysis and quantification of GLT-1 in WT-P_0 astrocytes, KO-P_0 astrocytes, and KO-P_0 astrocytes at 25 ng/mL concentration rmPEDF treatment. **F**, Gene expression of *serpinf1* mRNA in primary astrocyte transfected with PEDF-overexpress (PEDF-OE) or control plasmid (3 biological replicates). **G**, Western blotting analysis and quantification of GLT-1 and PEDF in primary astrocyte transfected with PEDF-OE or control plasmid. **H**, Western blotting analysis and quantification of GLT-1 in C6 at indicated concentration rmPEDF treatment. **I**, Western blotting analysis and quantification of GLT-1 and PEDF in C6 transfected with PEDF-OE or control plasmid. **J**, Gene expression of *serpinf1* mRNA in primary astrocyte transfected with si-*serpinf1* or si-NC (3 biological replicates). **K**, Representative images and quantification of GLT-1 co-stained with GFAP in primary cultured astrocyte transfected with si-*serpinf1* or si-NC. Data point represents primary astrocyte ($n = 8$ individual cells per group), from three independent experiments. Scale bars: 20 μm. Protein levels were normalized to β-actin, from three independent experiments. Data are presented as mean ± S.E.M. One-way ANOVA combined with the Tukey's multiple comparison (E), (H); Two-way ANOVA with Bonferroni's multiple-comparisons test (G), (I); Comparison by two-tailed unpaired

Student's *t*-test unless otherwise indicated. *$P < 0.05$, **$P < 0.01$, ***$P < 0.001$; n.s., not significant.

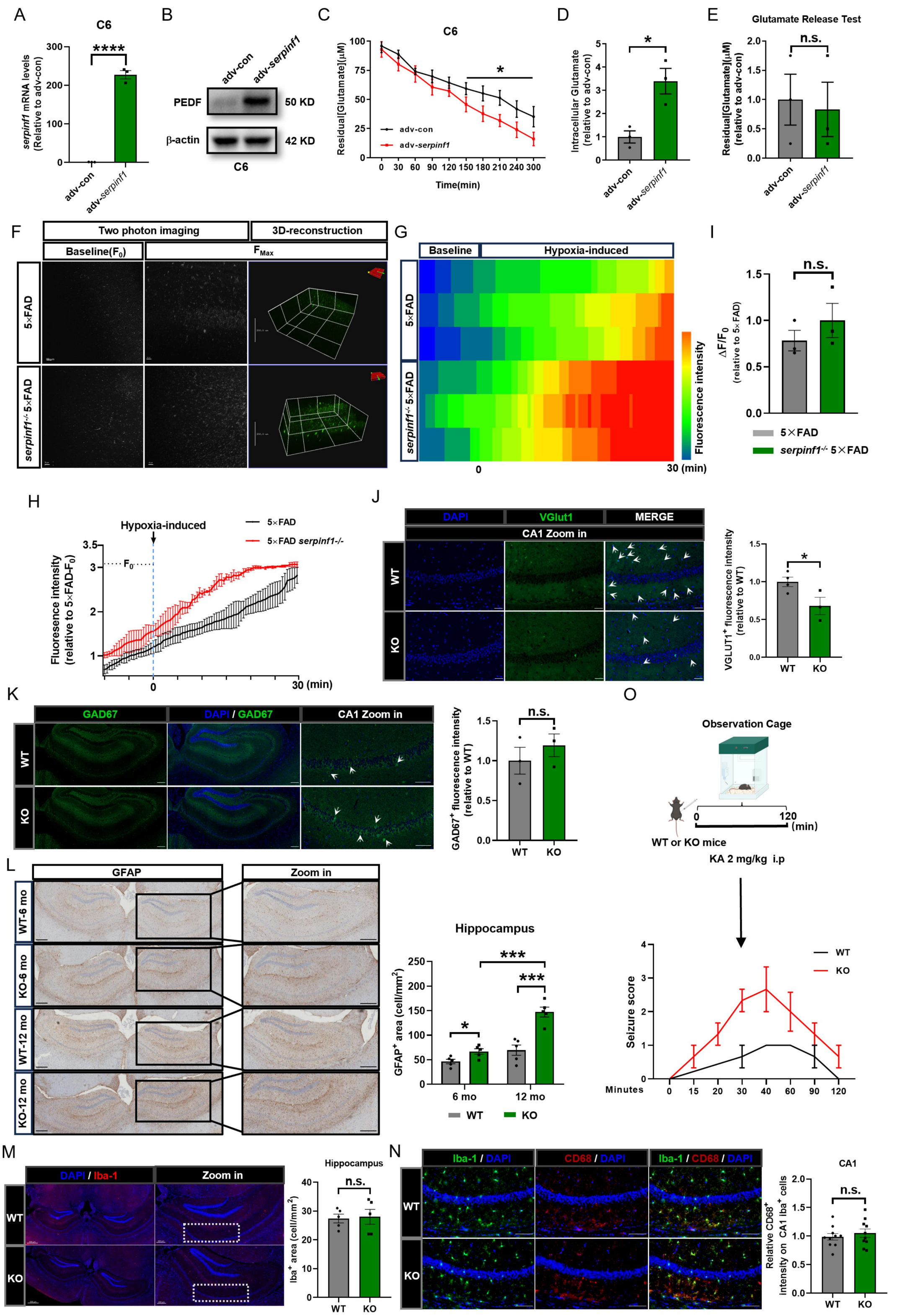

Figure S6. PEDF deficiency disrupts glutamate homeostasis AD mouse model and leads to abnormal activation of astrocytes. A-E, Gene expression of *serpinf1* mRNA (A), representative western blotting analysis of

PEDF (B), extracellular glutamate assay (C), intracellular glutamate assay (D) and glutamate release test (E) in C6 treated with PEDF-overexpress (adv-*serpinf1*) or control adenovirus (3 biological replicates). **F**, Representative 2D and 3D images of baseline and maximum glutamate signaling in the hippocampal slices of 5 × FAD and *serpinf1*$^{-/-}$ 5 × FAD mice. Scale bars: 20 μm (2D); 200 μm (3D). **G** and **H**, Representative heat map of average iGluSnFR intensity during the time course (G) and quantification of the average fluorescence intensity (H) (*n* = 3 mice per group). One‐way ANOVA with Fisher's LSD test. **I**, Quantification of pre- and post-hypoxia stimulated glutamate changes by the ratio of the difference between the baseline and maximum level of the glutamate signal to the baseline. **J** and **K**, Representative images and quantification of VGlut1 (J) and GAD67 (K) in the hippocampal CA1 of WT and KO mice (*n* = 3 mice per group). Scale bars: 200 μm (magnified view of CA1); 500 μm. **L** and **M**, Representative images and quantification of GFAP (L) or Iba1(M) in the hippocampal CA1 of KO and WT mice (*n* = 5 mice per group). Scale bars: 500 μm (magnified view of left panel); 1000 μm (left panel). **N**, Representative images and quantification of CD68 (activated microglia) co-stained with Iba1 (inactivated microglia) in the hippocampal CA1 of KO and WT mice (*n* = 10 individual cells from 5 mice per group). Scale bars: 200 μm. Data are presented as mean ± S.E.M. Two-way ANOVA combined with the Bonferroni's multiple-comparisons test (C), (H), (L), (O); Comparison by two-tailed unpaired Student's *t*-test unless otherwise

indicated. *$P < 0.05$, ***$P < 0.001$, ****$P < 0.0001$; n.s., not significant.

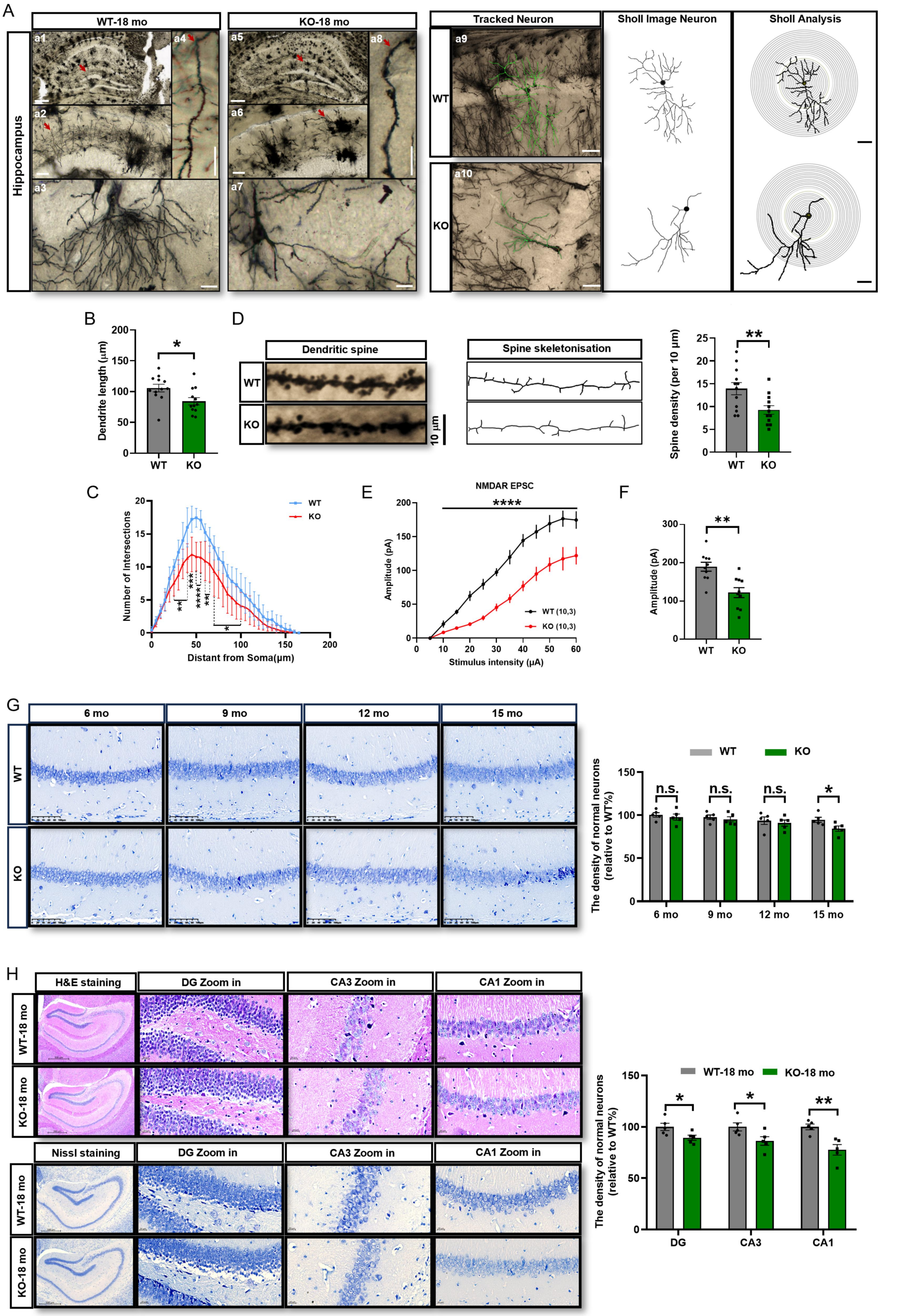

Figure S7. Pathological changes in dendritic spine morphology and neuronal loss in PEDF-deficient mice. A, Representative confocal micrographs of the morphology of pyramidal neurons in the hippocampus of

18-mo-old WT and KO mice (left panel). 3D neuron tracking reconstruction and Sholl analysis images (right panel). Scale bar: 500 μm (a1, a5), 200 μm (a2, a6), 50 μm (a3, a7, Track Neuron), 20 μm (a4, a8). **B** and **C**, Total cumulative lengths of basal dendritic processes neuron (b) and Sholl analysis of dendritic arbors of pyramidal neurons (c) in the hippocampus (n = 12 cells from 3 individual mice per group). **D**, Representative confocal micrographs and spines-skeletonized images of dendritic spines of pyramidal neurons (left panel). Scale bar: 10 μm. Quantitative analysis of dendritic spine density (n = 12 cells from 3 individual mice per group) (right panel). **E** and **F**, Recording (E) and quantitative analysis (F) of NMDAR-EPSC amplitude in hippocampal CA1 pyramidal neurons of 6-mo-old WT and KO mice (n = 10 cells from 3 individual mice per group). **G**, Representative images of Nissl staining of the hippocampal CA1 of WT and KO mice (left panel); quantification of the density of normal neurons (right panel) (n = 5 mice per group). Scale bar: 100 μm. **H**, Representative images of H&E and Nissl staining of the hippocampal of 18-mo-old WT and KO mice (left panel); quantification of the density of normal neurons (right panel) (n = 5 mice per group). Scale bar: 20 μm (magnified view); 500 μm. Data are presented as mean ± S.E.M. Two-way ANOVA combined with the Bonferroni's multiple-comparisons test (C), (E); Comparison by two-tailed unpaired Student's t-test unless otherwise indicated. *P < 0.05, **P < 0.01, ***P < 0.001, ****P < 0.0001; n.s., not significant.

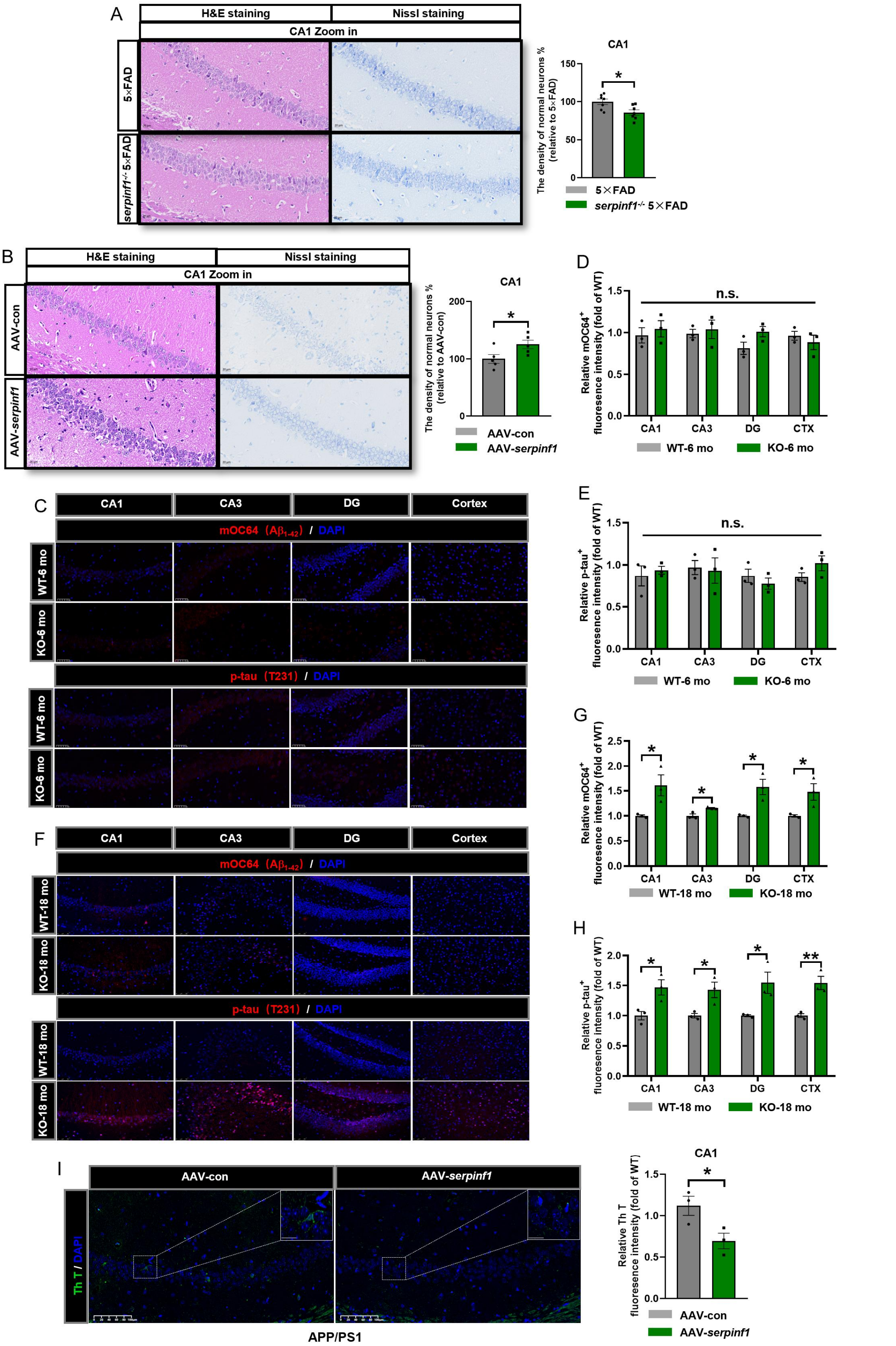

Figure S8. PEDF deficiency increases AD-related pathological burden. A and B, Representative images of H&E and Nissl staining of the hippocampal CA1 of 8-week-old 5 × FAD and *serpinf1*$^{-/-}$ 5 × FAD mice (A) or 6-mo-old

AAV-con and AAV-*serpinf1* injected mice (B); quantification of the density of normal neurons (n = 5 mice per group). Scale bar: 20 μm. **C-H**, Representative images of $A\beta_{1-42}$ and phosphorylated-tau (p-tau) staining of the hippocampal CA1 of 6-mo-old (C) or 18-mo-old (F) KO and WT mice; quantification of $A\beta_{1-42}$ and p-tau immunofluorescence intensity of 6-mo-old (D and E) or 18-mo-old (G and H) KO and WT mice (n = 3 mice per group). Scale bar: 50 μm. **I**, Representative images (left panel) and quantification of the density (right panel) of Thioflavin-T (Th T) staining of the hippocampal CA1 of AAV-con and AAV-*serpinf1* injected mice. Scale bar: 20 μm (magnified view); 100 μm. Data are presented as mean ± S.E.M. Comparison by two-tailed unpaired Student's t-test unless otherwise indicated. *P < 0.05, **P < 0.01; n.s., not significant.

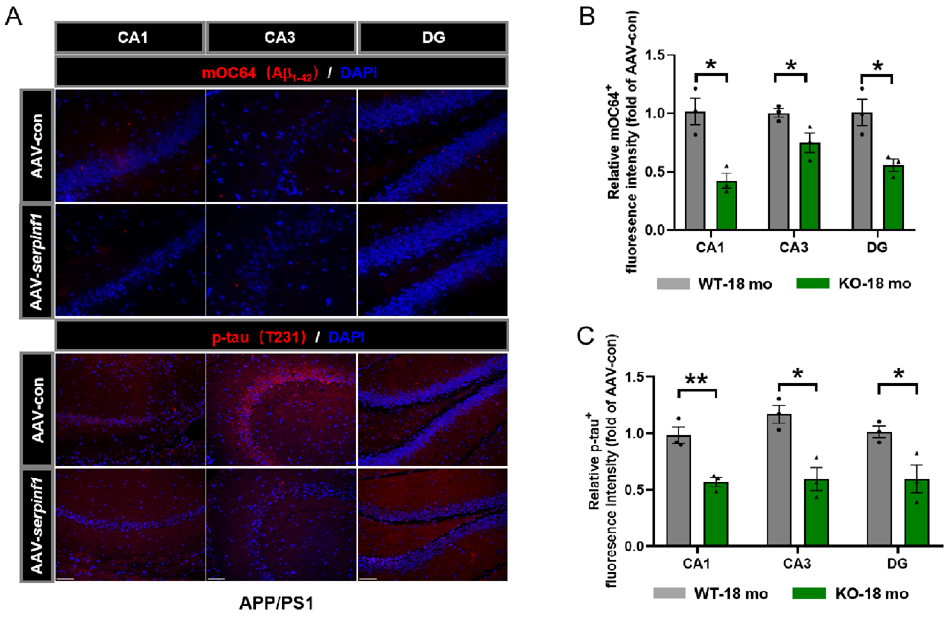

Figure S9. Restoration of PEDF attenuates amyloid and tau pathology in an AD model. A-C, Representative images of Aβ_{1-42} and phosphorylated-tau (p-tau) staining of the hippocampal of 6-mo-old AAV-con and AAV-*serpinf1* injected mice (A); quantification of A β $_{1-42}$ (B) and p-tau (C) immunofluorescence intensity (n = 3 mice per group). Scale bar: 50 μm. Data are presented as mean ± S.E.M. Comparison by two-tailed unpaired Student's *t*-test unless otherwise indicated. **P* < 0.05, ***P* < 0.01.

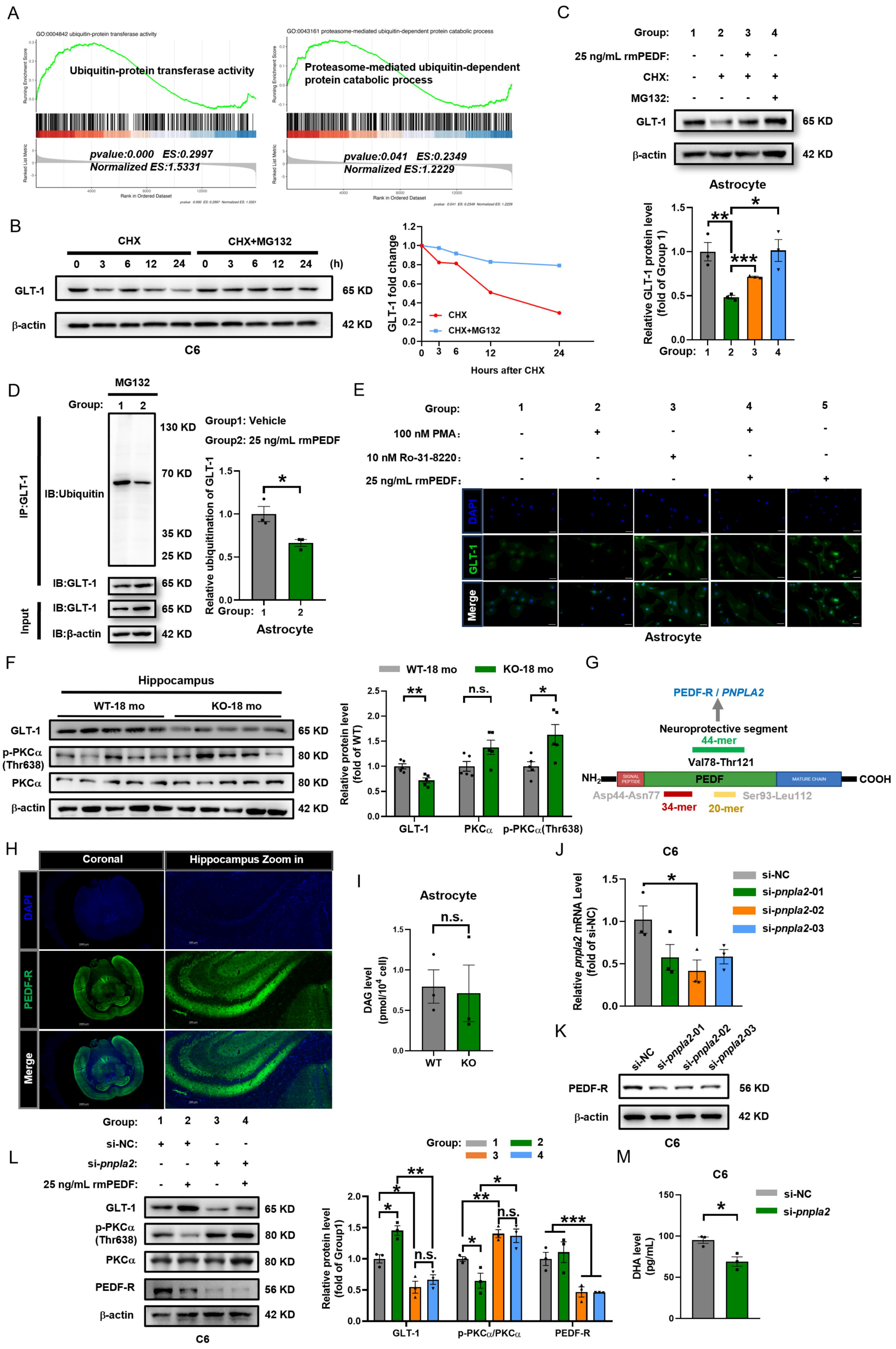

Figure S10. PEDF deficiency increases PKC α phosphorylation *via* blockade of PEDF/PEDF-R signaling, resulting in excessive degradation

of astrocytic GLT-1. A, GSEA of ubiquitin-protein transferase activity, proteasome-mediated ubiquitin-dependent protein catabolic process hallmark pathway. **B**, Western blotting analysis and quantification of GLT-1 in C6 incubated in 100 μg/mL CHX with or without MG132 treatment at indicated time points. **C**, Western blotting analysis of and quantification of GLT-1 in astrocytes treated with rmPEDF (25 ng/mL), CHX (100 μg/mL), MG132 (10 μM), or both for 24 h, as indicated. **D**, Astrocytes were transfected with vehicle or rmPEDF (25 ng/mL) in the presence of MG132. GLT-1 was immunoprecipitated (IP). Western blotting analysis and quantification of ubiquitinated proteins or GLT-1. **E**, Representative images of GLT-1 staining astrocytes treated with rmPEDF (25 ng/mL), PMA (100 nM), Ro-31-8220 (10 nM), or both for 24 h, as indicated. Scale bar: 50 μm. **F**, Representative western blotting analysis and quantification of GLT-1, PKCα, and p-PKCα in the hippocampal of 18-month-old WT and KO mice ($n = 5$ mice per group). **G**, Schematic of the structure of the PEDF protein. **H**, Representative images of PEDF-R staining in the brain section of C57B6/J mice. 100 μm (magnified view); 1000 μm. **I**, DAG level in WT and KO-P_0 astrocytes (3 biological replicates). **J** and **K**, Gene expression of *pnpla2* mRNA (J) and representative western blotting analysis (K) of PEDF-R in control (si-NC), PEDF-R silenced (si-*pnpla2*) C6. **L**, Western blotting analysis and quantification of GLT-1, PEDF-R, PKCα and p-PKCα in C6 treated with si-NC, si-*pnpla2* and rmPEDF (25 ng/mL), or both for 24 h, as indicated. **M**, DHA level in C6 treated with

si-NC and si-*pnpla2*. Protein levels were normalized to β-actin, from three independent experiments. Data are presented as mean ± S.E.M. One-way ANOVA combined with the Tukey's multiple comparison (C), (J); Two-way ANOVA with Bonferroni's multiple-comparisons test (B), (L); Comparison by two-tailed unpaired Student's *t*-test unless otherwise indicated. *$P < 0.05$, **$P < 0.01$, ***$P < 0.001$; n.s., not significant.

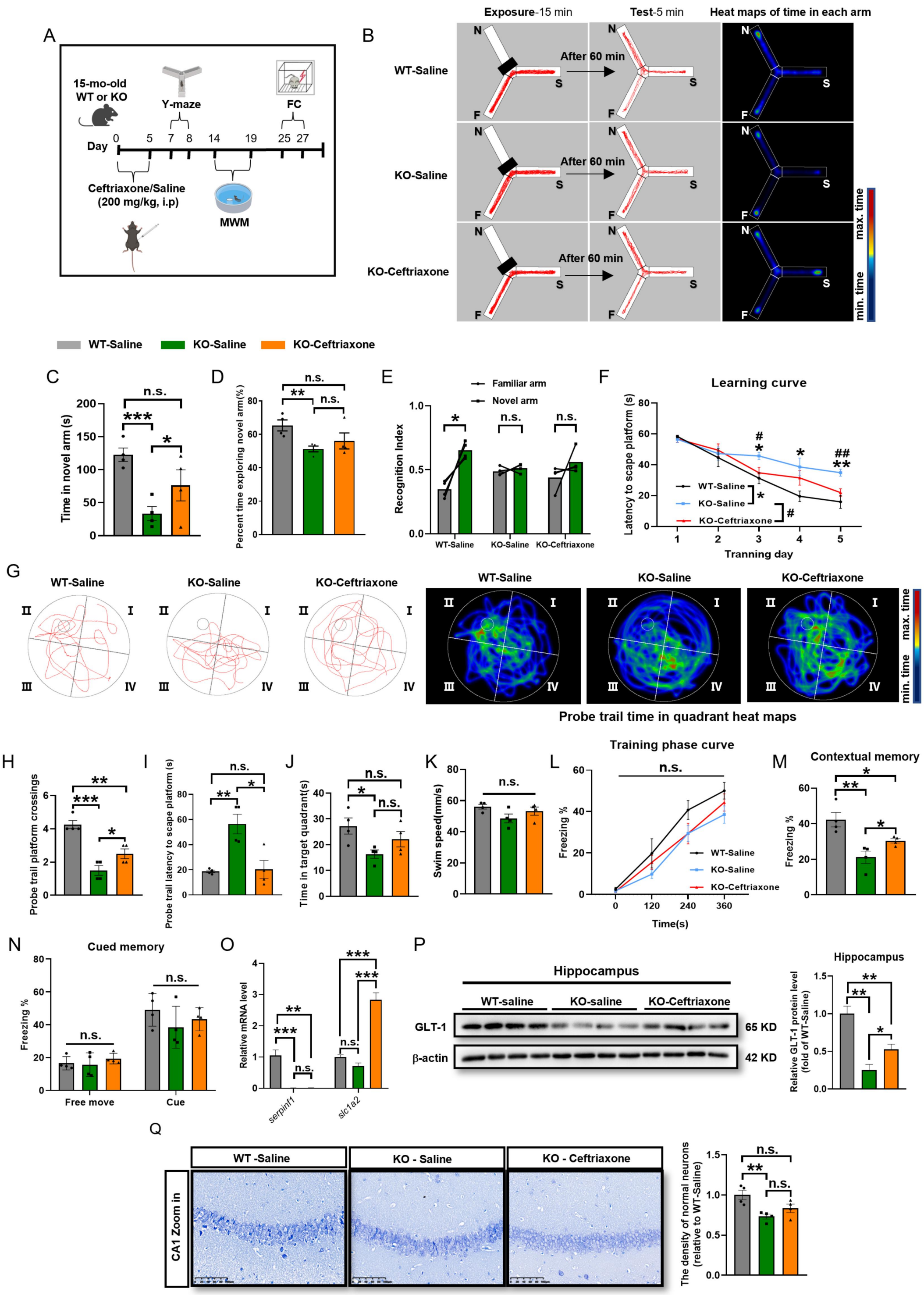

Figure S11. Ceftriaxone partially rescues cognitive deficits in PEDF-deficient mice. A, Schematic diagram showing the Ceftriaxone treatment strategy in 15-mo-old WT or KO mice. **B-E**, Representative track and heat map images (B), time spent in novel arm (C), percent time exploring

the novel arm to total time exploring novel arm and familiar arm (D), discrimination index (E) by WT or KO mice treated with saline or Ceftriaxone in the Y-maze test. **F**, Time to reach hidden platform in MWM plotted across training days. **G-K**, Representative track and heat map images of mice (G), time spent in target quadrant (H), platform crossing count (I), latency of first time to enter the target (J) and mean swimming speed (K) in the probe trial of the MWM test. **L-N**, Percent time of freezing in training phase (L), contextual memory test (M) and cued memory test in FC test (N). **O** and **P**, Gene expression of *sepinf1* and *slc1a2* mRNA (O), representative western blotting analysis and quantification (P) of GLT-1 in WT or KO mice treated with saline or Ceftriaxone. **Q**, Representative images of Nissl staining of the hippocampal CA1 of WT or KO mice treated with saline or Ceftriaxone (left panel); quantification of the density of normal neurons (right panel) (n = 4 mice per group). Scale bar: 100 μm. Data are presented as mean ± S.E.M. One-way ANOVA combined with the Tukey's multiple comparison (C), (D), (H-K), (M), (P), (Q); Two-way ANOVA with Bonferroni's multiple-comparisons test (F), (L), (N), (O); Comparison by two-tailed unpaired Student's t-test unless otherwise indicated. *$P < 0.05$, **$P < 0.01$, ***$P < 0.001$; n.s., not significant.

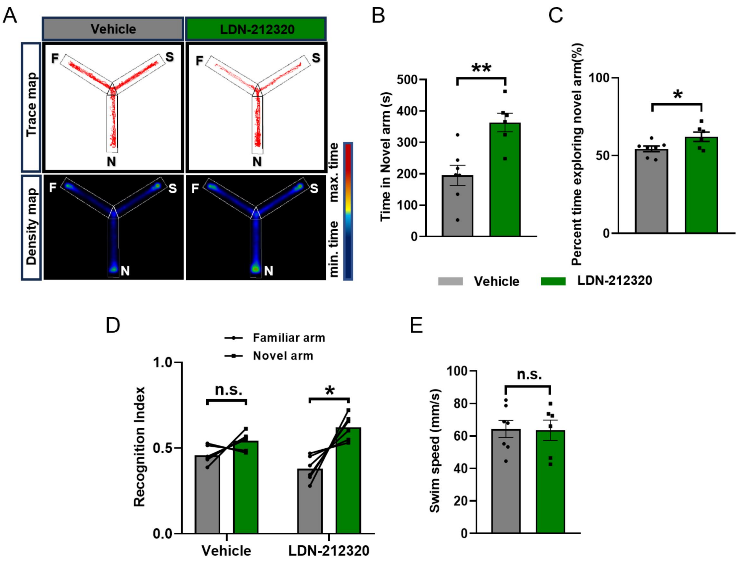

Figure S12. LDN-212320 rescues cognitive deficits in PEDF-deficient mice. A-D, Representative track and heat map images (A), time spent in novel arm (B), percent time exploring the novel arm to total time exploring novel arm and familiar arm (C), discrimination index (D) in the Y-maze test (*n* = 7 mice per group). **E**, Mean swimming speed by KO mice treated with vehicle and LDN-212320 in the probe trial of the MWM test. Data are presented as mean ± S.E.M. Two-tailed paired Student's *t*-test (D). Comparison by two-tailed unpaired Student's *t*-test unless otherwise indicated. **P* < 0.05, ***P* < 0.01; n.s., not significant.

Supplemental Tables:

Supplemental Table 1. Participants' demographics and clinical characteristics

**Characteristic**	**Non-dementia**	**Dementia**	***p* value**
N	75	85 (including *n* = 19 AD samples)	N/A
Male	34	37	N/A
Female	41	48	N/A
Age (years)	55-90	60-95	N/A
Average age (years)	75.17 ± 2.22	72.46 ± 9.53	0.59
On-set age (years)	N/A	62.38	N/A
Disease duration (months)	N/A	27.52	N/A

p value: Non-dementia vs. Dementia.

N/A, Not Available; All data are all presented as the mean ± SD.

Supplemental Table 2. KEGG analysis of the Top 50 proteins in PPI analysis

Term ID	Background gene count	FDR	Matching proteins in network	Strength	Term description
mmu05010	359	0.0149	16	0.51	**Alzheimer disease**
mmu05012	239	0.0149	13	0.6	Parkinson disease
mmu05020	264	0.0354	12	0.52	Prion disease
mmu00190	130	0.0149	9	0.7	Oxidative phosphorylation

Supplemental Table 3. The list of antibodies used in this paper

Antibodies	Source	Catalog	Application	Dilution
anti-β-actin	Sigma	A5441	WB	1:5000
anti-PEDF	Millipore	MAB-1059	WB/IF	1:1000 / 1:100
anti-GLT-1	Abcam	Ab41621	IF	1:200
anti-GLT-1	Santa Cruz Biotechnology	Sc-365634	WB	1:2000
anti-GLAST	Santa Cruz Biotechnology	Sc-515839	WB	1:2000
anti-GS	Abcam	ab228590	WB	1:2000
anti-EAAC1	Abways Techology	CY8613	WB	1:2000
anti-GFAP	Cell Signaling Technology	3670S	IF	1:500
anti-Iba-1	Wako	019-19741	IF	1:200
anti-VGLUT1	Abclonal	A12879	IF	1:100
anti-GAD67	Abclonal	A2938	IF	1:100
anti-β-Amyloid$_{1-42}$(mOC64)	Abcam	Ab201060	IF	1:100
anti- p-tau (T231)	Abcam	Ab151559	IF	1:100
anti-PSD-95	Santa Cruz Biotechnology	Sc-32290	WB	1:1000
anti-Synaptophysin	Santa Cruz Biotechnology	Sc-55507	WB	1:1000
anti-Caspase-3	Cell Signaling Technology	9662s	IF	1:200
anti-Ubiquitin	Cell Signaling Technology	3933	WB	1:2000

anti-Pan p-Ser/Thr/Tyr	Abmart	M210030	WB	1:1000
anti-PKCα	Cell Signaling Technology	2056S	WB	1:2000
anti-p-PKCα (Thr638)	Cell Signaling Technology	9375s	WB	1:1000
anti-PEDF-R	Cayman	10006409	WB/IF	1:2000 / 1:100
DAPI	Sigma	D8417	IF	1:1000
Anti-rabbit	Vector Laboratory	PI-1000	WB	1:2000
Anti-mouse	Vector Laboratory	PI-2000	WB	1:2000
488-Donkey anti-rabbit	ThermoFisher	A32731	IF	1:200
594-Donkey anti-mouse	ThermoFisher	A21203	IF	1:200

316

Supplemental Table 4. Oligonucleotide sequence used for qPCR primers

**Primer name**	**Primer Sequences (5’ to 3’)**
m-s*erpinf1*-Forward	CCAACTTCGGCTACGATCTGT
m-s*erpinf1*-Reverse	TCTGTTCGATGTTCAGCTCCC
m-*slc1a2*-Forward	GCCAACAATATGCCCAAGCAG
m-s*lc1a2*-Reverse	GACACCAAACACAGTCAGTGA
m-a*ctb*-Forward	CACTGTCGAGTCGCGTCC
m-a*ctb*-Reverse	CCAGTTGGTAACAATGCCATGT
rat-*pnpla2*-Forward	TGGATGAAGGAGCAGACAGGTAGC
rat-*pnpla2*-Reverse	AGTGGCACAGACGGCAGAGAC
rat-a*ctb*-Forward	GAGAGGGAAATCGTGCGTGA
rat-a*ctb*-Reverse	CAGGGAGGAAGAGGATGCGG

Supplemental Table 5. Abbreviations in this paper

Abbreviation	Full name
AD	Alzheimer's disease
SERPINs	Serine protease inhibitors
CSF	Cerebrospinal Fluid
$5 \times$ FAD	Transgenic mouse model carrying five familial Alzheimer's disease mutations (B6SJL-Tg (APPSwFlLon, PSEN1*M146L*L286V)6799Vas/Mmjax)
APP/PS1	Transgenic mice expressing a chimeric mouse/human amyloid precursor protein (Mo/HuAPP695swe) and a mutant human presenilin 1 (PS1-dE9)
SERPINF1	Serine protease inhibitor F1
PEDF	Pigment epithelium-derived factor
RNA-seq	RNA sequencing
MMSE	Mini-Mental State Examination
MoCA	Montreal Cognitive Assessment
CB	Cerebellum
MY	Medulla
TH	Thalamus
OLF	Olfactory cortical areas
HPF	Hippocampus
WB	Western blotting
KO	Knockout
RT-qPCR	Real-time quantitative PCR
IF	Immunofluorescence
OFT	Open field test
NOR	Novel object recognition
MWM	Morris water maze

PCA	Principle-component analysis
DEGs	Differentially expressed genes
KEGG	Kyoto Encyclopedia of Genes and Genomes
GSEA	Gene-set enrichment analysis
GO	Gene Ontology
PPI	Protein-protein interaction
AAV	Adeno-associatedvirus
BP	Biological process
CC	Cellular compartment
MF	Molecular function
EAAT1	Excitatory amino acid transporter 1
GS	Glutamine synthetase
GLAST	Glutamate-L-aspartate transporter
EAAC1	Excitatory amino acid carrier 1
OE	Overexpression
NMDAR	N-methyl-D-aspartate receptor
fEPSP	Field excitatory postsynaptic potential
EPSCs	Excitatory postsynaptic currents
LTP	Long-term potentiation
CHX	Cycloheximide
Co-IP	Co-immunoprecipitation
PMA	Phorbol 12-myristate 13-acetate
CEF	Ceftriaxone
PKCα	Protein kinase C alpha
KA	Kainic acid
Co-IP	co-immunoprecipitation
ATGL	Adipose Triglyceride Lipase
DAG	Diacylglycerol
PLA_2	Phospholipase A_2

DHA	Docosahexaenoic acid
ALS	Amyotrophic lateral sclerosis
CNS	Central nervous system
HBSS	Hank's Balanced Salt Solution
PS	Pen-strep
RT	Room temperature
ATCC	American Type Culture Collection
SDS	Sodium dodecyl sulfate
